# Different *MAPT* haplotypes influence expression of *total MAPT* in postmortem brain tissue

**DOI:** 10.1186/s40478-023-01534-9

**Published:** 2023-03-11

**Authors:** Christina V. Tauber, Sigrid C. Schwarz, Thomas W. Rösler, Thomas Arzberger, Steve Gentleman, Otto Windl, Mandy Krumbiegel, André Reis, Viktoria C. Ruf, Jochen Herms, Günter U. Höglinger

**Affiliations:** 1 Department of Translational Neurodegeneration, German Center for Neurodegenerative Diseases (DZNE), Munich, Germany; 2 Department of Neurology, School of Medicine, Technical University Munich, Munich, Germany; 3 Department of Obstetrics and Gynecology, Ludiwgs-Maximilians University of Munich, Munich, Germany; 4 Center for Neuropathology and Prion Research, Ludwig-Maximilians University of Munich, Munich, Germany; 5 Department of Psychiatry and Psychotherapy, University Hospital, Ludwig-Maximilians-University, Munich, Germany; 6 Parkinson’s UK Brain Bank, Department of Brain Sciences, Imperial College London, London, UK; 7 Neuropathology Unit, Department of Brain Sciences, Department of Medicine, Imperial College London, London, UK; 8 Institute of Human Genetics, Friedrich-Alexander University of Erlangen-Nuremberg, Erlangen, Germany; 9 Munich Cluster for Systems Neurology (SyNergy), Munich, Germany; 10 Department of Neurology, Ludwig-Maximilians University of Munich, Munich, Germany

**Keywords:** Parkinson’s disease, *MAPT*, Tau protein, H1 and H2 haplotype, Synucleins, Postmortem human brain, Gene expression, Protein level

## Abstract

The *MAPT* gene, encoding the microtubule-associated protein tau on chromosome 17q21.31, is result of an inversion polymorphism, leading to two allelic variants (H1 and H2). Homozygosity for the more common haplotype H1 is associated with an increased risk for several tauopathies, but also for the synucleinopathy Parkinson’s disease (PD). In the present study, we aimed to clarify whether the *MAPT* haplotype influences expression of *MAPT* and *SNCA*, encoding the protein α-synuclein (α-syn), on mRNA and protein levels in postmortem brains of PD patients and controls. We also investigated mRNA expression of several other *MAPT* haplotype-encoded genes. Postmortem tissues from cortex of fusiform gyrus (ctx-fg) and of the cerebellar hemisphere (ctx-cbl) of neuropathologically confirmed PD patients (*n* = 95) and age- and sex-matched controls (*n* = 81) were *MAPT* haplotype genotyped to identify cases homozygous for either H1 or H2. Relative expression of genes was quantified using real-time qPCR; soluble and insoluble protein levels of tau and α-syn were determined by Western blotting. Homozygosity for H1 versus H2 was associated with increased *total MAPT* mRNA expression in ctx-fg regardless of disease state. Inversely, H2 homozygosity was associated with markedly increased expression of the corresponding antisense *MAPT-AS1* in ctx-cbl. PD patients had higher levels of insoluble 0N3R and 1N4R tau isoforms regardless of the *MAPT* genotype. The increased presence of insoluble α-syn in PD patients in ctx-fg validated the selected postmortem brain tissue. Our findings in this small, but well controlled cohort of PD and controls support a putative biological relevance of tau in PD. However, we did not identify any link between the disease-predisposing H1/H1 associated overexpression of *MAPT* with PD status. Further studies are required to gain a deeper understanding of the potential regulatory role of *MAPT-AS1* and its association to the disease-protective H2/H2 condition in the context of PD.

**Supplementary Information:**

The online version contains supplementary material available at 10.1186/s40478-023-01534-9.

## Introduction

Parkinson’s disease (PD) is the second most common neurodegenerative disease after Alzheimer’s disease (AD). PD affects more than 6 million people worldwide [1]. The major neuropathologic hallmarks of PD are the loss of dopaminergic neurons in the substantia nigra and the accumulation of cytoplasmic inclusions, so-called Lewy bodies (LB), composed primarily of the protein α-synuclein (α-syn) [2]. In the past 2 decades, genome-wide association studies (GWAS) have identifyed genetic susceptibility loci that confer the risk for sporadic PD [3]. The strongest genetic associations are common variations at the *SNCA* gene, encoding α-syn on chromosome 4, followed by the H1/H1 haplotype on Ch17q21.31 that includes *MAPT*, encoding for the microtubule-associated protein tau [4–9] and several other genes.

Tau is primarily expressed in the central nervous system [10–13]. Six tau isoforms are present in the human brain, which differ by expression of either 3 or 4 microtubule binding domain repeats (termed 3R or 4R isoforms) and the number of N-terminal inserts (termed 0N, 1N, 2N isoforms) [14]. Pathologic aggregates containing hyperphosphorylated tau protein occur within the brain in a variety of neurodegenerative diseases termed tauopathies, including AD, corticobasal degeneration (CBD) and progressive supranuclear palsy (PSP). The latter diseases CBD and PSP are clinically characterized as atypical Parkinson syndromes [15]. In PD, aggregated tau can also be found colocalized to a certain extent with the aggregates composed primarily of α-syn [16–18]. In vitro studies demonstrated that tau and α-syn can mutually promote each others aggregation [19, 20].

The *MAPT* gene is located within a 952 kb haplotype block on chromosome 17q21.31, which is the result of an inversion polymorphism, leading to two allelic variants, called H1 and H2 haplotypes [21, 22]. H1 homozygosity (H1/H1) is more common in the human population and associated with an increased risk for tauopathies like AD, CBD and PSP [23–28], but surprisingly also for the synucleinopathy PD [6, 8, 29–32]. Inversely, H2 homozygosity (H2/H2) is considered to be protective against tauopathies [23–27] as well as PD [33]. Studies on postmortem brain and human neural cells suggested that H1 homozygosity is associated with an increased *MAPT* expression and different expression patterns of *MAPT* transcripts on mRNA level [34, 35]. Increased mRNA levels of 4R *MAPT* [36], but lower levels of 2N *MAPT* transcripts, have been described for H1/H1 compared to H2/H2 in 10 different postmortem brain regions of 134 healthy subjects [37]. The mRNA ratio of 4R to 3R *MAPT* was found to be increased in the cerebellum of PD cases not genotyped for *MAPT* haplotype [14]. These findings imply that haplotype- and disease-specific expression and splicing of *MAPT* may contribute to the association of H1-homozygosity with sporadic PD.

Notably, the *MAPT* block not only comprises the *MAPT* gene, but 19 other nucleotide sequences. Among these, a number of transcripts have been shown to be associated with PD. Similar to various long non-coding antisense genes [38, 39], a differential expression profile in PD was identified for *MAPT-AS1* [40] and it might be involved in PD by altering the expression of PD-related genes [41]. The the intronless gene *STH* with unknown protein function may be involved in the regulation of 3R and 4R *MAPT* splicing [34]. Located just outside the region of the H1/H1 haplotype, the gene *NSF* was identified as risk factor for PD by GWAS studies [42]. It is involved in synaptic vesicle transport [42, 43] which is considered one of the major pathways implicated in PD etiology [44]. For other transcripts in the H1/H1 *MAPT* haplotype, like *PLEKHM1*, also involved in intracellular vesicle processes [45], no association with PD has been found to date.

In summary, these prior studies described differences in gene expression at the *MAPT* locus in human brains, but never in the context of PD and *MAPT* haplotype simultaneously. Some publications compared *MAPT* haplotypes of healthy donors [34–37], others only PD versus controls without haplotype differentiation [14]. Therefore, we investigated the influence of both the *MAPT* haplotype and the PD status on mRNA expression levels of *MAPT*, *MAPT-AS1, STH, PLEKHM1* and *NSF* within the *MAPT* locus as well as the alpha-, beta-, gamma-synuclein genes *SNCA, SNCB, SNCG.* In addition, we examined protein levels of soluble and insoluble tau and α-syn under the same paradigm. The analysis was performed in *MAPT* genotyped human postmortem samples of the cortex of fusiform gyrus (ctx-fg), a brain region typically affected by α-syn pathology in PD [46], and the cortex of cerebellum (ctx-cbl), a region whithout α-syn pathology in PD.

## Materials and methods

### Human postmortem brain tissue

**Fig. 1:**
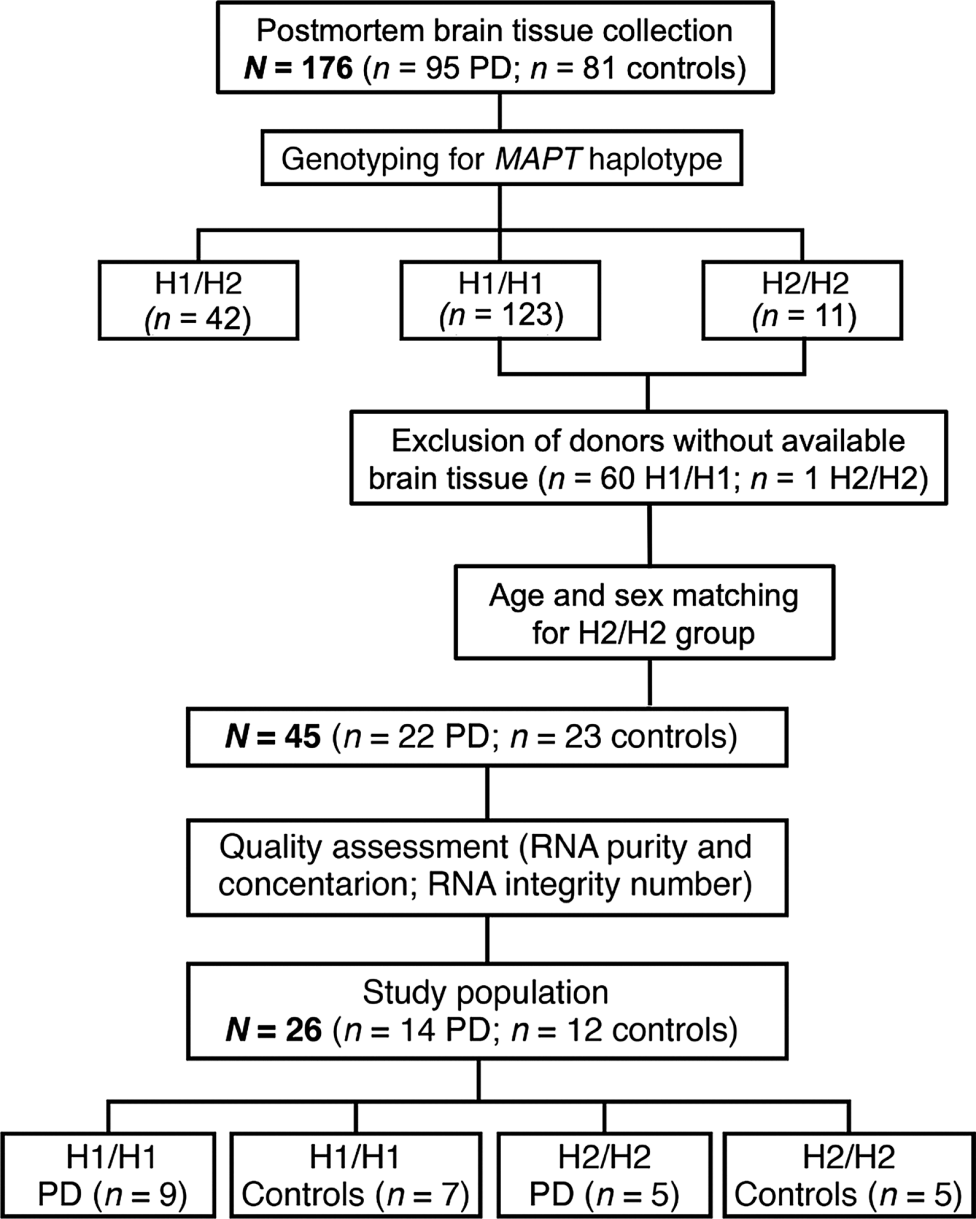
Selection of the human postmortem brain samples. Postmortem brain tissue was obtained from sporadic Parkinson’s disease (PD) patients and healthy controls. After genotyping for *MAPT* haplotype, neuropathological validation, and tissue quality control, suitable samples from two brain regions [cortex of fusiform gyrus (PD-affected region), cortex of cerebellum (unaffected control region)] were allocated in four age- and sex-matched groups, defined by the *MAPT* haplotype (H1/H1, H2/H2) and disease status (PD, controls)

Human postmortem brain tissue was obtained from the Neurobiobank of the Ludwig-Maximilians-University of Munich (*n* = 92) and the Parkinson’s UK Brain Bank (*n* = 84). A multi-step selection process was used to identify the best cases for the study population (Fig. 1). The specimen ultimately included derived from clinically well documented and neuropathologically confirmed cases of PD and healthy controls. Donors were collected in accordance with the requirements of the local Ethics committee of the brain banks. Either the donors had given their full consent to the use of their brains for research during lifetime or the relatives consented in accordance with the presumed will of the deceased. Our analyses have been approved by the Research Ethics committee of the Technical University Munich (Nr. 265/16 S) and Imperial College London (18/WA/0238). For exclusion of high tau burden AD Braak and Braak scores [47] were assessed by board-certified neuropathologists. Exclusion criteria for control and PD cases were either severe concomitant tau pathology (> AD Braak and Braak stage 3), clinical report of dementia or positive family history of PD, or a postmortem interval (PMI) from death to tissue fixation of more than 50 h. Inclusion criteria for PD cases was Lewy Body Disease (LBD) Braak stage > 3 [46]. Additionally, all cases were assessed according to the CERAD score (The Consortium to Establish a Registry for Alzheimer's Disease) [48] and PD cases according to the McKeith classification [49]. Presence of α-syn pathology was confirmed for PD donors by semi-quantitative evaluation in the target brain region ctx-fg through an experienced neuropathologist (Additional file 2: Figure S1). Comprehensive neuropathological reports were available for all included cases, and clinical data from medical records were gathered, as far as available (Additional file 1: Table S1). The causes of death were also reported (Additional file 1: Table S2). Since sex-related differences in alterations of mRNA expression associated with PD have been described, sex matching was done for the final study population [50, 51]. Two types of tissue were included in this study: ctx-fg, as pathologic region typically affected by LB pathology from LBD Braak stage 3 on, and ctx-cbl, a region without α-syn pathology, as control region. Besides the high availability in brain banks, the ctx-fg was chosen because it is not severely affected by neurodegeneration despite being affected early with α-syn inclusion pathology. Tissue blocks of both brain regions were macroscopically dissected in grey and white matter, fresh frozen and stored at − 80 °C. The frozen specimens were thawed as briefly as necessary and then cut lying on dry ice. The dissection of the specimens described here refers to both ctx-fg and ctx-cbl. During the dissection of the cerebellum samples, the separation from the white matter was not always clearly possible due to the foliae. To focus on neuronal enrichment only, grey matter was used for mRNA expression and protein level analysis.

### Genotyping and tissue selection

In total, brain tissue of 176 donors (PD *n* = 95, control *n* = 81) were collected for *MAPT* H1/H2 genotyping. DNA extraction was performed using the Blood & Tissue DNA Extraction kit (Qiagen, Venlo, The Netherlands). In cooperation with the Institute of Human Genetics, Friedrich-Alexander University of Erlangen-Nürnberg (Erlangen, Germany), four haplotype tagging single-nucleotide-polymorphisms (SNP) were determined (rs17650901, rs1052553, rs9468, rs8070723) using Taq DNA polymerase followed by BigDye Terminator v.3.1 Cycle Sequencing Kit with 3730 Genetic Analyzer (Thermo Scientific, Waltham, USA). PCR primers used for genotyping are listed in Additional file 1: Table S3.

### RNA extraction, quality criteria and cDNA synthesis

In order to prevent RNA degradation during experimental workflow, all materials and surfaces were cleaned using RNase Zap™ (Sigma-Aldrich, St. Louis, MO, USA) and samples were constantly kept on dry ice. Fresh frozen brain tissue was initially homogenized with a tissue grinder, Potter–Elvehjem type, (VWR, Radnor, USA) in QIAzol Lysis Reagent (Qiagen) followed by gDNA elimination solution and phase separation with chloroform and centrifugation in MaXtract High Density Tubes (Qiagen). Subsequent column purification was performed using the RNEasy + Universal Mini Kit (Qiagen) according to the manufacturer’s instructions. RNA concentration and purity were quantified using a spectrophotometer (NanoDrop™ 2000c, Thermo Scientific). As thresholds a ratio of A_260_/A_230_ > 1.5 and A_260_/A_280_ > 1.8 for purity and > 0.3 µg/µl for concentration were chosen. RNA quality criteria were analyzed in 45 preselected cases and led to the exclusion of 5 tissue donors. RNA integrity (RIN) was verified using the BioAnalyzer (Agilent Technologies, Santa Clara, USA). If necessary, samples were diluted to fit the chip’s working range of 50–500 ng/µL. RIN was measured in 40 preselected tissue donors in both brain regions, from which the samples with the best value were selected for the study population (exclusion *n* = 14 donors). RIN values ranged from 2.8 to 8.1 and did not differ between subgroups in ctx-fg or ctx-cbl (Additional file 1: Table S4). One µg of total RNA was used for reverse transcription with the iScriptTM cDNA synthesis kit (Bio-Rad Laboratories, Inc., Hercules, USA) following the manufacturer’s instruction. cDNAs were stored afterwards at − 20 °C until use.

### Quantiative real-time PCR

Semi quantitative real time PCR (qPCR) was carried out using the StepOne Plus Real-time PCR System (Applied Biosystems). Reactions were performed using the SYBR Green Master Mix (Applied Biosystems) in a 20 µL reaction volume, containing 10 µL SYBR Green Master Mix (Sigma-Aldrich), 1 µL of each primer, 4.3 µL cDNA and 6.8 μL water. The following program parameters were used for all amplifications: 95 °C for 10 min, 40 cycles at 95 °C for 15 s and 60 °C for 1 min, followed by 95 °C for 15 s and 60 °C for 1 min. All assays were performed using three technical replicates. A cDNA standard serial dilution was prepared from a pool of sample cDNA and was run for each primer on every plate. Relative mRNA expression levels from samples were calculated with this relative standard curve to ensure comparability of samples between plates. For evaluation of reference gene expression stability, 17 candidate reference genes were tested previously in postmortem brain tissue. Expression stability and optimal number of reference genes required for normalization of the respective setup were estimated using the geNorm algorithm (Biogazelle, Gent, Belgium). Proposed threshold values for appropriate gene stability in a heterogeneous setup, such as clinical biopsies or postmortem tissue, are means of the gene stability measure (M) < 1 and the coefficient of variation (CV) < 0.5 [52]. The most stable reference genes (*TBP, ACTIN, GAPDH*) resulted in M = 1.037 and CV = 0.427 for both brain regions and were used as internal control. Primer specificity was previously confirmed with 3% agarose gel electrophoresis for all used primers. Primers for 0N, 1N and 2N *MAPT* were designed based on the publication of Spicakova et al. [53]. Full primer sequences are shown in Additional file 1: Table S5. Upon revision it was noted that the used primer for detection of the *NSF* gene covers *NSF, NSF* pseudogene and *LRRC37A2.* Of note, the NSF pseudogene is also expressed in human brain according to Ensembl database (www.ensemble.org) [54]. Pseudogenes are considered non-functional, yet there is evidence of potential regulatory function at RNA level i.e. in tumor biology [55]. Similar to a study investigating the link between single nucleotide polymorphisms in the *NSF* gene and cocain dependence in peripheral blood samples, specific gene expression of certain NSF variants failed due to the genetic overlap of NSF and the NSF pseudogene [56]. The gene *LRRC37A2* is located in a large copy number variation within NSF exons [57] and was considered negligible in this context with its expression primarily in testis and not in human brain (proteinatlas.org). Taken together, the primer used in our study did not specifically detect the *NSF* gene and it cannot be excluded that the co-detection of the *NSF* pseudogene had an impact on the, albeit non-significant, result in our study.

The real time-qPCR data were analyzed using the qBase + 3.2 software (Biogazelle). Normalization of target genes was conducted using the determined reference genes. Fold change was calculated by scaling calibrated normalized relative quantitites of mRNA expression to the reference group and values were log2 transformed for each gene analyzed per brain region. The subgroup H2/H2 control was choosen as reference group since H2/H2 is assumed to be the protective variant in terms of PD development. When performing the two-way ANOVA, the H2/H2 control samples were included in the H2/H2 group for analysis of main effects "effect of haplotype" and "effect of disease" and interaction haplotype x disease. Candidate genes were selected which had not yet been studied in depth or which are of great interest in PD. In total, expression levels of 5 genes were investigated encoded in the *MAPT* inversion region (*total MAPT* and *MAPT transcripts, MAPT-AS1, NSF, PLEKHM1, STH*) in the study population.

### Protein extraction and Western blot

Sequential protein extractions were performed from the very same tissue block used for mRNA expression. Two different protein fractions (high-salt-buffer-soluble as “soluble” and sarkosyl-soluble as “insoluble”) were obtained using a protocol adapted from the publication of Strauß et al. [58]. Thus, fresh frozen brain tissue was homogenized in 4 volumes of high salt-buffer (50 mM Tris–HCl pH 7.4, 750 mM Sodium Chloride, 10 mM Sodium Fluoride, 5 mM EDTA, Sigma-Aldrich) supplemented with protease inhibitors (cOmplete, Roche Basel, Switzerland) and phosphatase inhibitors (PhoSTOP™, Roche). After 20 min incubation, homogenates were ultracentrifuged in Ultra-Clear Centrifuge Tubes (Beckman Coulter™, Brea, USA) at 100,000 g for 30 min at 4 °C. The supernatant was reserved as the high salt buffer soluble protein fraction. For insoluble protein extraction with sarkosyl, the remaining pellet was washed once with 400 µl high salt-buffer and centrifuged at 100,000 g for 30 min at 4 °C. Pellets were incubated overnight in 2 volumes of sarkosyl-buffer (High Salt Buffer supplemented with 1% sarkosyl (Sigma-Aldrich) in an end-over-end rotator. The samples were again ultracentrifuged at 100,000 g for 30 min at 4 °C and the supernatant was collected as insoluble protein fraction (sarkosyl soluble fraction). Protein concentrations were measured by Pierce™ Bicinchoninic Acid Assay Kit (Thermo Scientific) assay. For analysis of tau, equal amounts of protein were dephosphorylated using λ protein phosphatase (New England Biolabs, Ipswich, USA) at a final concentration of 0.3 U/µL for 3 h at 30 °C. 20 µg of protein were denatured with XT-Buffer (75 °C for 10 min) and loaded on (α-syn: 10%; tau: 12%) Bis–Tris Criterion polyacrylamide gels (Bio-Rad) and electrophoresis was run with MES running buffer (α-syn: 140 V for 70 min; tau: 160 V for 125 min). Proteins were blotted on a 0.2 µm PVDF membrane for 40 min at 1 A and 25 V with transfer buffer containing 20% methanol. After the transfer, the membrane was fixed in 0.4% paraformaldehyde in PBS for 20 min. After washing 3 × with PBS, the membrane was blocked with 5% skimmed milk (Sigma-Aldrich) in TBS supplemented with 0.05% Tween-20 (TBS-T, Sigma-Aldrich) for α-syn immunoblotting, and with 3 × ROTI®Block (Carl Roth, Karlsruhe, Germany) for tau immunoblotting, for at least 1 h and then incubated overnight at 4° C with the primary antibodies. The membrane was washed and incubated with secondary peroxidase-linked antibodies for 2 h at room temperature. The blots were developed with Clarity Western ECL Substrate (Bio-Rad Laboratories) and the signal was detected with Odyssey Fc imaging system (LI-COR Biotechnology, Lincols, Netherlands). After imaging, the blots were analyzed with the Empiria StudioTM Software from LI-COR. Applied antibodies were diluted in 1 × ROTI®Block in TBS-T (Carl Roth) and are listed in Additional file 1: Table S6. For stripping, the membrane was rinsed after imaging with TBS-T and then incubated in stripping buffer for 20 min at 50 °C. Thereafter, the membrane was washed thoroughly first with deionized water and then with TBS-T before blocking again with milk and 3 × ROTI®Block respectively. The incubation with primary and secondary antibodies as well as the imaging was performed as described above. The protein concentration was determined, and 20 μg protein was loaded on every SDS gel together with the samples of interest for the soluble and the insoluble fraction, respectively. All blots were run in triplets. Due to the predetermined slots on pre-casted gels it was not possible to accommodate all samples of the study population per protein of interest on one blot. Tau blots included one set of representative samples of combination of haplotype and disease, while α-syn blots fitted two sets. In addition, we used one additional lane with the very same postmortem protein pool in all blots as inter- run calibration control and quality control. These were generated by pooling some microliter of all samples from the high salt buffer-soluble samples and the sarkosyl-soluble samples, respectively. For inter-run calibration, normalization was also performed for the pool of all samples represented on all blots (for soluble and insoluble). The respective quality control displayed only minor variability between blots (< 15%) garantueeing a valid comparison and analysis of several gels. Secondly, the signals from every western blot were normalized to the signal from the reference protein of the same sample (soluble protein: GAPDH; insoluble protein: RevertTM 700 Total Protein Stain). Total tau was determined as the sum of the individual isoforms. Specific tau isoforms were identified by comparison to the recombinant tau ladder. Full length Western Blots used for representative figures are displayed in Additional file 2: Figure S2-S5.

### Statistics

Statistical analysis was performed using Graphpad Prism 9.2.0 for Mac (GraphPad Software Inc., San Diego, CA, USA). For characterization of the study cohort, one-way ANOVA for quality markers such as age at death, mean RIN and mean PMI was performed. For the prevalence of *MAPT* haplotypes within the study cohort a chi-squared test was conducted. For mRNA expression and protein level data, two-way ANOVA was conducted investigating the effect of disease (PD vs. control), of the *MAPT* haplotype (H1/H1 vs. H2/H2), and their interaction on the dependent variables. Correction for multiple testing was performed with Tukey’s post hoc test. Normality was assessed using Shapiro–Wilk test and homogeneity of variances was evaluated with Levene’s test. Data was analyzed separately in ctx-gf and ctx-cbl, as ratio of ctx-gf to ctx-cbl, and ratio of *MAPT isoform*/*total MAPT*, tau isoform/total tau respectively. All pairwise comparisons were run with reported 95% confidence intervals and *P* values were Bonferroni-adjusted. For all statistical tests the significance level was set to *P* < 0.05. Specifications are given in each figure and table legend. Data are presented as *mean* ± *SEM*, unless indicated otherwise.

## Results

### Selection of postmortem brain tissues

A sample of postmortem brains, including 95 PD cases and 81 controls without neurodegenerative diseases, has been genotyped for the *MAPT* haplotype (Fig. 1). The H1/H1 haplotype constellation was most frequent (~ 70%), followed by H1/H2 (~ 24%), and the rare H2/H2 haplotype (~ 6%) (Table 1). In our sample, the PD disease status and the *MAPT* haplotype were not significantly associated, most likely due to our sample size being too small to address this specific issue. Sufficient brain tissue for our biochemical analyses was available from 115 cases. Next, brains with *MAPT* H1/H1 genotypes were matched for age and sex to the H2/H2 brains and RNA quality criteria (RNA purity, concentration and integrity number). At the end of the selection process 15% of the samples were included into the study. The final study sample consisted of 14 PD brains [*n* = 9 (64%) with *MAPT* H1/H1, *n* = 5 (36%) with H2/H2] and 12 controls [*n* = 7 (58%) with *MAPT* H1/H1, *n* = 5 (42%) with H2/H2) (Fig. 1, Table 2).

**Table 1:** Frequency of *MAPT* haplotypes determined in postmortem brain donors

*MAPT* haplotype	Controls		PD		PD versus controls
	(*n* = 81)		(*n* = 95)		*P* value
H1/H1 [*n* (%)]	55	(67.9)	68	(71.6)	0.596
H1/H2 [*n* (%)]	21	(25.9)	21	(22.1)	0.553
H2/H2 [*n* (%)]	5	(6.2)	6	(6.3)	0.968

*P* values were calculated using Chi-squared test

**Table 2:** Demographic, clinical and neuropathologic characteristics of postmortem brain donors

	Controls		PD		Group-wise comparison
	H1/H1	H2/H2	H1/H1	H2/H2	*P* value
	(*n* = 7)	(*n* = 5)	(*n* = 9)	(*n* = 5)	
Sex (female) [*n* (%)]	4 (57.1)	4 (80.0)	4 (44.4)	3 (60.0)	0.380^†^
Age at death (y) [*mean* (SD)]	74.0 (6.8)	81.0 (15.2)	74.2 (3.2)	77.6 (7.2)	0.433^#^
PMI (h) [*mean* (SD)]	15.7 (7.5)	25.3 (9.2)	15.9 (5.9)	15.7 (9.1)	0.145^#^
Age at diagnosis (y) [*mean* (SD)]	–	–	64.3 (6.5)	68.0 (7.8)	0.415^‡^
Disease duration (y) [*mean* (SD)]	–	–	9.0 (7.4)	10.25 (3.4)	0.760^‡^
Lewy inclusion pathology in ctx-fg					0.536*
None [*n* (%)]	–	–	1 (11.1	1 (20.0)	
Few [*n* (%)]	–	–	4 (44.4)	3 (60.0)	
Moderate [*n* (%)]	–	–	3 (33.3)	–	
Many [*n* (%)]	–	–	1 (11.1)	1 (20.0)	
LBD Braak stage					0.504*
1 [*n* (%)]	–	–	–	–	
2 [*n* (%)]	–	–	–	–	
3 [*n* (%)]	–	–	–	–	
4 [*n* (%)]	–	–	1 (11.1)	1 (16.7)	7
5 [*n* (%)]	–	–	2 (22.2)	–	
6 [*n* (%)]	–	–	6 (66.7)	4 (83.3)	
LBD McKeith stage					0.472*
Neocortical [*n* (%)]	–	–	5 (55.6)	4 (80.0)	
Limbic [*n* (%)]	–	–	2 (22.2)	–	
Brain stem predominant [*n* (%)]	–	–	1 (11.1)	1 (20.0)	
AD Braak and Braak stage					0.363*
I [*n* (%)]	3 (42.9)	3 (75.0)	4 (44.4)	4 (80.0)	
II [*n* (%)]	4 (57.1)	–	4 (44.4)	1 (20.0)	
III [*n* (%)]	–	1 (25.0)	1 (11.1)	–	
IV [*n* (%)]	–	–	–	–	
V [*n* (%)]	–	–	–	–	
VI [*n* (%)]	–	–	–	–	
CERAD score					0.280*
0 [*n* (%)]	7 (100.0)	5 (100.0)	8 (88.9)	3 (60.0)	
A [*n* (%)]	–	–	–	2 (40.0)	
B [*n* (%)]	–	–	1 (11.1)	–	
C [*n* (%)]	–	–	–	–	

The LBD Braak stage and AD Braak and Braak stage consist of six stages, each. The CERAD score describes neuritic Amyloid-ß plaques in levels of 0, A, B, C. Lewy inclusion pathology assessed semi-quantitatively the burden of Lewy neurites and Lewy bodies in the target brain region cortex of fusiform gyrus. For group-wise comparisons of the subgroups, defined by *MAPT* haplotype and disease status, *P* values were calculated by: *, Chi-squared test; #, one-way ANOVA; ‡, unpaired Student’s t-test; †, Fisher’s exact test

AD, Alzheimer’s disease; CERAD, The Consortium to Establish a Registry for Alzheimer's Disease; ctx-fg, cortex of fusiform gyrus; LBD, Lewy body disease; PD, Parkinson’s disease; PMI, postmortem interval

### Characteristics of study sample

The sex distribution was 15:11 female to male in the total study population and did not differ between the PD and control subgroups. There were no significant differences in demographic, clinical or neuropathologic characteristics between the groups defined by disease status (PD, Con) and genotype (H1/H1, H2/H2) (Table 2). The severity of α-syn pathology in the PD cases was overall high with a median LBD Braak stage of 6 (of max. 6) in both H1/H1 and H2/H2 PD brains, with no difference between both groups. Presence of α-syn pathology was confirmed in the target brain region, i.e. ctx-fg, of most PD donors (86%) by semi-quantitative evaluation of Lewy inclusion pathology (Lewy neurites and LB) with no difference between H1/H1 and H2/H2 PD cases (Table 2, Additional file 1: Table S1). The pattern of LB pathology according to the McKeith classification [49] were distributed among PD cases as neocortical (*n* = 9), brain stem predominant (*n* = 2), limbic (*n* = 1), and limbic/neocortical (*n* = 1). Controls had neither clinical Parkinsonism during lifetime, nor any LB pathology. The burden of tau pathology was equally low with only two cases of AD Braak score III (H1/H1 PD *n* = 1; H2/H2 Control* n* = 1). Neither did subgroups of PD and controls differ significantly regarding concomitant AD pathology in terms of ß-amyloid neuritic plaques (CERAD score, Table 2).

### Influence of the PD disease status and MAPT haplotype on mRNA expression

**Fig. 2:**
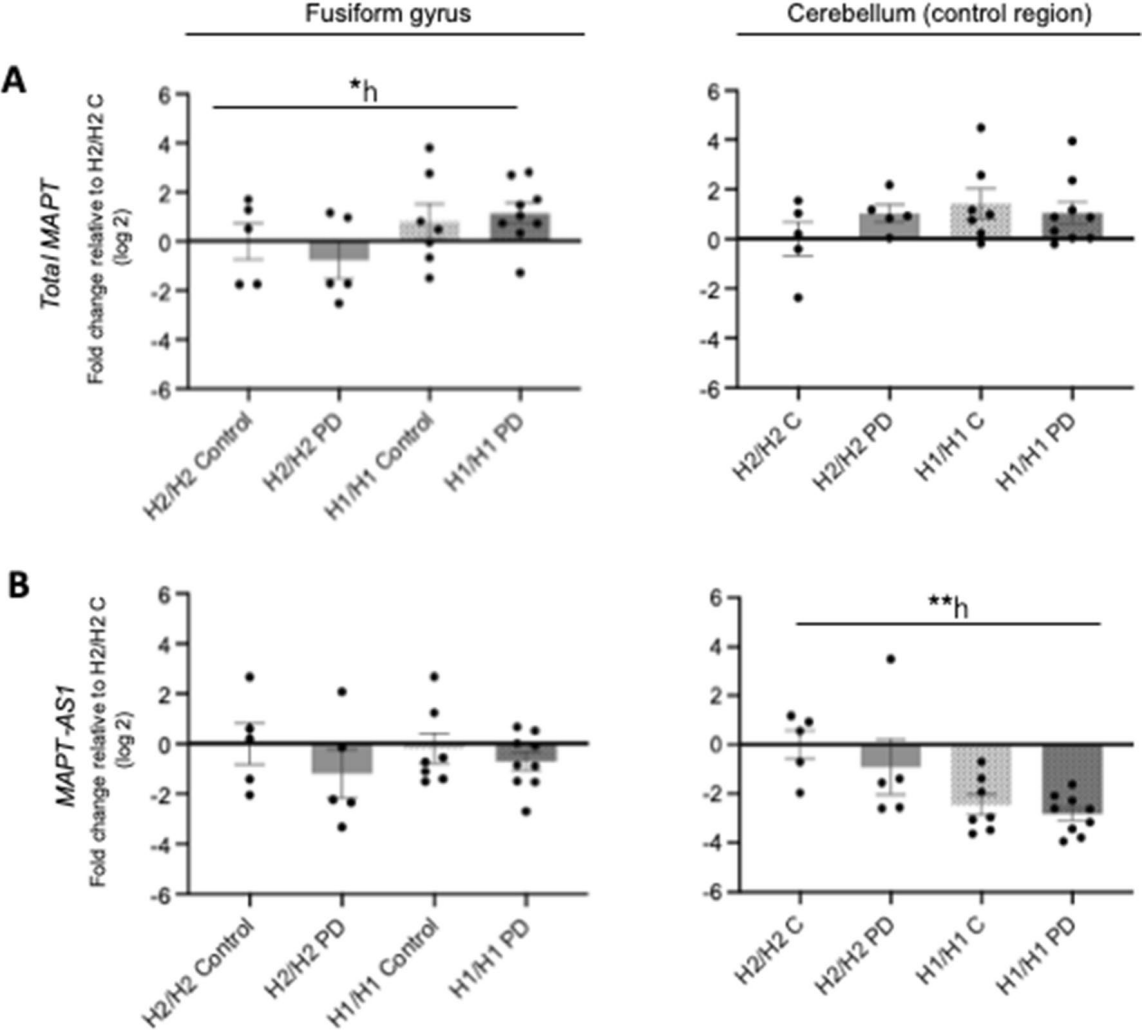
Influence of *MAPT* haplotype and disease status on *MAPT* and *MAPT-AS1* mRNA expression. Bar graphs showing the effect of *MAPT* haplotype (H1/H1, H2/H2) and diseases status (PD, controls) on the mRNA expression of *total MAPT* (**A**) and *MAPT-AS1* (**B**) measured by qPCR in human brain samples of the cortex of fusiform gyrus and the cortex of cerebellum. For analysis of the *MAPT* haplotype effects, PD cases and controls were combined, and vice versa for analysis of the disease status. Data are fold change (log2, mean ± SEM) relative to H2/H2 controls (H2/H2 C). The total independent sample size is: H2/H2 Control *n* = 5, H2/H2 PD *n* = 5, H1/H1 Control *n* = 7, H1/H1 PD *n* = 9). Two-way ANOVA (haplotype × disease status), followed by Tukey’s post-hoc test: *P < 0.05; **P < 0.01; *h, significant effect of haplotype

As shown by qPCR, the PD disease status had no effect on the expression of *total MAPT, MAPT* splice variant transcripts *0N, 1N, 2N, 3R* and *4R MAPT*, *MAPT-AS1* and the synuclein genes *SNCA, SNCB* and *SNCG*, neither in ctx-fg nor in ctx-cbl (Fig. 2, Table 3).

**Table 3:** Effects of *MAPT* haplotype and disease status on expression of candidate mRNAs in human samples of fusiform gyrus and cerebellum

mRNA	Brain region	Two-way ANOVA	Main effect of haplotype	Main effect of disease	Haplotype × disease	Direction of significant effect
		*P* value	*P* value	*P* value	*P* value	
*Total MAPT*	ctx-fg	0.182	**0.047**	0.741	0.404	H1/H1 > H2/H2
	ctx-cbl	0.365	0.147	0.698	0.725	–
*0N MAPT*	ctx-fg	0.367	0.546	0.331	0.123	–
	ctx-cbl	0.614	0.228	0.672	0.710	–
*1N MAPT*	ctx-fg	0.162	0.229	0.538	0.062	–
	ctx-cbl	0.534	0.151	0.988	0.910	–
*2N MAPT*	ctx-fg	0.540	0.781	0.651	0.671	–
	ctx-cbl	0.155	0.703	0.196	0.130	–
*3R MAPT*	ctx-fg	0.290	0.752	0.541	0.300	–
	ctx-cbl	0.614	0.938	0.244	0.743	–
*4R MAPT*	ctx-fg	0.338	0.896	0.809	0.369	–
	ctx-cbl	0.652	0.796	0.149	0.316	–
*MAPT-AS1*	ctx-fg	0.617	0.822	0.201	0.600	–
	ctx-cbl	0.007	**0.011**	0.272	0.647	H2/H2 > H1/H1
*STH*	ctx-fg	0.594	0.728	0.813	0.568	–
	ctx-cbl	0.458	0.170	0.646	0.432	–
*NSF*	ctx-fg	0.745	0.285	0.615	0.953	–
	ctx-cbl	0.822	0.442	0.618	0.915	–
*PLEKHM1*	ctx-fg	0.487	0.544	0.162	0.535	–
	ctx-cbl	0.497	0.467	0.519	0.101	–
*SNCA*	ctx-fg	0.640	0.777	0.214	0.661	–
	ctx-cbl	0.730	0.533	0.679	0.438	–
*SNCB*	ctx-fg	0.325	0.242	0.159	0.874	–
	ctx-cbl	0.388	0.488	0.915	0.143	–
*SNCG*	ctx-fg	0.839	0.768	0.407	0.753	–
	ctx-cbl	0.916	0.556	0.923	0.626	–

Bold values indicate a significant result with *P* < 0.05

Main effect of haplotype was analyzed by comparison of H1/H1 (Controls + PD) versus H2/H2 (Controls + PD). Main effect of disease was analyzed by comparison of PD (H1/H1 + H2/H2) versus Controls (H1/H1 + H2/H2). The interaction haplotype × disease examined the relationship between the independent variables (haplotype + disease) on the dependent variable of mRNA expression. *P* values were calculated using two-way ANOVA followed by Tukey’s multiple comparisons test

ctx-fg, cortex of fusiform gyrus; ctx-cbl, cerebellum

However, the *MAPT* haplotype did influence mRNA expression of *total MAPT*, with significantly higher expression in H1/H1 (~ twofold) compared to H2/H2 in ctx-fg (F (1,22) = 4.41, *P* = 0.047), but not in ctx-cbl (Fig. 2, Table 3). For the *MAPT* splice variant transcripts, no significant differences were found between both haplotypes in both brain regions (Table 3). Also, the expression of *MAPT* splice variants relative to *total MAPT* expression was neither affected by disease, nor *MAPT* haplotype, nor by their combination in both analyzed brain regions (Additional file 1: Table S7). The transcription profile of *MAPT-antisense 1* (*MAPT-AS1*) was significantly lower in H1/H1 (-2.6 fold) compared to H2/H2 (F (1.22) = 14.21, *P* = 0.001) in ctx-cbl, but not in ctx-fg (Fig. 2, Table 3). As expected, the *MAPT* haplotype did not affect synuclein (*SNCA*, *SNCB*, *SNCG*) mRNA expression (Table 3).

The mRNA expression of *PLEKHM1*, *NSF* and *STH* within the *MAPT* inversion region were not statistically significant different neither between H1/H1 and H2/H2 carriers, nor between control and PD cases, in both brain regions (Table 3). The expression levels of all examined transcripts were not affected by an interaction haplotype × disease (two-way ANOVA, Fig. 2, Table 3). Furthermore, mRNA expression in ctx-fg did not differ significantly from expression levels in ctx-cbl for all analyzed transcripts (Additional file 1: Table S8).

### No influence of the PD disease status and MAPT haplotype on soluble protein levels

**Fig. 3:**
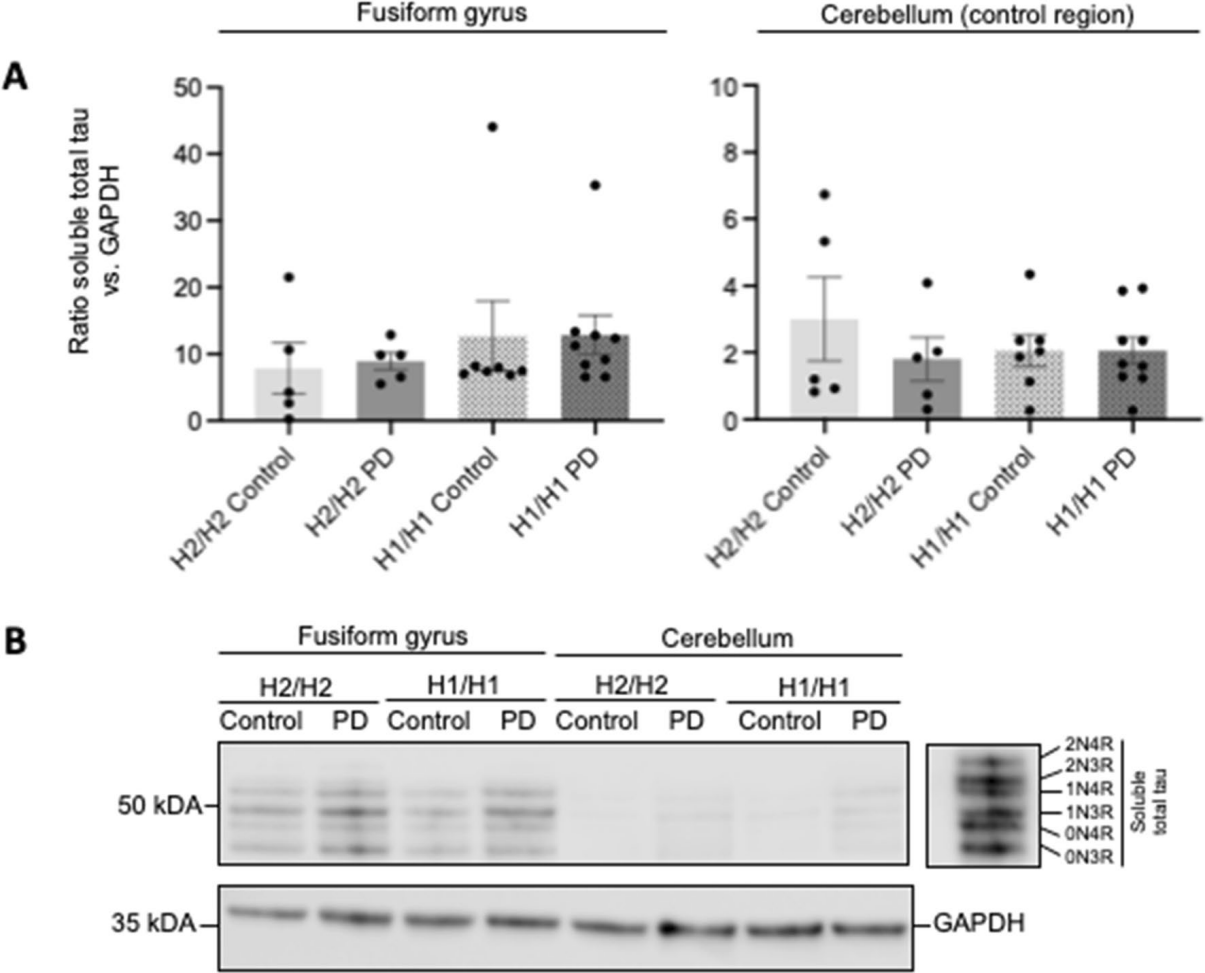
Effect of *MAPT* haplotype and disease status on soluble tau protein levels. **A** Bar graphs showing the effect of *MAPT* haplotypes (H1/H1, H2/H2) and diseases status (PD, control) on soluble total tau protein levels in human brain samples of cortex of fusiform gyrus and the cortex of cerebellum. For analysis of the *MAPT* haplotype effects, PD cases and controls were combined, and vice versa for analysis of the disease status. Data are protein quantity (mean ± SEM) relative to GAPDH and normalized to an inter-run calibration consisting of pooled protein samples. Two-way ANOVA (haplotype × disease status) showed no significant group differences. **B** Representative Western blots for soluble total tau with GAPDH as reference protein. The six tau isoforms were identified by comparison to a recombinant tau ladder. Each group is represented with 1 donor in each blot. The total independent sample size is: H2/H2 Control *n* = 5, H2/H2 PD *n* = 5, H1/H1 Control *n* = 7, H1/H1 PD *n* = 9). Full length Western blots are shown in Additional file 2: Figure S2

**Fig. 4:**
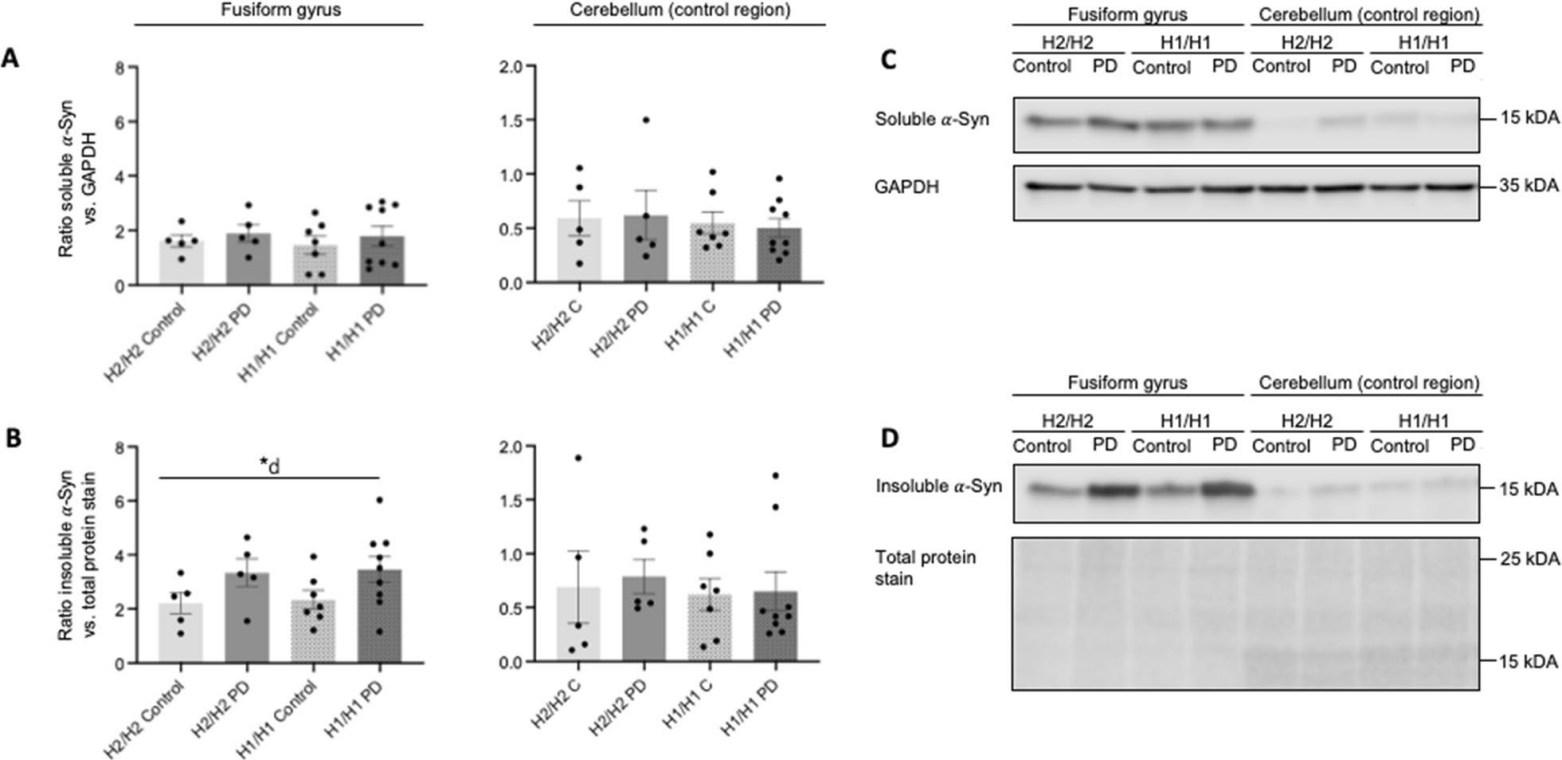
Influence of *MAPT* haplotype and disease status on soluble and insoluble α-synuclein protein levels. Bar graphs showing the effect of *MAPT* haplotypes (H1/H1, H2/H2) and diseases status (PD, control) on protein levels of soluble (**A**) and insoluble (**B**) α-synuclein (α-Syn) in human brain samples of the cortex of fusiform gyrus and the cortex of cerebellum. For analysis of the *MAPT* haplotype effects, PD cases and controls were combined, and vice versa for analysis of the disease status. Data are protein quantity (mean ± SEM) relative to GAPDH and normalized to an inter-run calibration consisting of pooled protein samples. Two-way ANOVA (haplotype × disease status), followed by Tukey’s post-hoc test: *P < 0.05; *d, significant effect of disease. **C**, **D** Representative Western blot for soluble (**C**) and insoluble (**D**) α-Syn with GAPDH (**C**) and Total Protein Stain (**D**) as reference. Each group is represented with 1 donor in each blot. The total independent sample size is: H2/H2 Control *n* = 5, H2/H2 PD *n* = 5, H1/H1 Control *n* = 7, H1/H1 PD *n* = 9). Full length Western blots are shown in Additional file 2: Figures S3 and S4

Neither disease nor *MAPT* haplotypes nor their interaction influenced the protein levels of total tau, its isoforms, or α-syn in the high-salt-buffer-soluble protein fraction in both analyzed brain regions (Figs. 3, 4A, Table 4).

**Table 4:** Effects of *MAPT* haplotype and disease status on levels of tau isoforms and α-syn in the soluble protein fraction in human samples of fusiform gyrus and cerebellum

Protein fraction	Brain region	Two-way ANOVA	Main effect of haplotype	Main effect of disease	Haplotype × disease	Direction of significant effect
		*P* value	*P* value	*P* value	*P* value	
α-syn	ctx-fg	0.830	0.595	0.511	0.899	–
	ctx-cbl	0.926	0.563	0.946	0.790	–
Total tau	ctx-fg	0.737	0.279	0.877	0.908	–
	ctx-cbl	0.285	0.415	0.875	0.077	–
0N3R tau	ctx-fg	0.312	0.101	0.406	0.734	–
	ctx-cbl	0.207	0.207	0.150	0.205	–
0N4R tau	ctx-fg	0.341	0.103	0.479	0.724	–
	ctx-cbl	0.301	0.176	0.332	0.251	–
1N3R tau	ctx-fg	0.308	0.093	0.455	0.721	–
	ctx-cbl	0.412	0.223	0.519	0.263	–
1N4R tau	ctx-fg	0.446	0.145	0.552	0.700	–
	ctx-cbl	0.480	0.898	0.919	0.132	–
2N3R tau	ctx-fg	0.538	0.653	0.184	0.551	–
	ctx-cbl	0.581	0.251	0.574	0.610	–
2N4R tau	ctx-fg	0.709	0.316	0.607	0.749	–
	ctx-cbl	0.072	0.569	0.482	0.052	–

Main effect of haplotype was analyzed by comparison of H1/H1 (Controls + PD) versus H2/H2 (Controls + PD). Main effect of disease was analyzed by comparison of PD (H1/H1 + H2/H2) versus Controls (H1/H1 + H2/H2). The interaction haplotype × disease examined the relationship between the independent variables (haplotype + disease) on the dependent variable of protein levels. *P* values were calculated using two-way ANOVA followed by Tukey’s multiple comparisons test

ctx-fg, cortex of fusiform gyrus; ctx-cbl, cerebellum.

### Influence of the PD disease staus and MAPT haplotype on insoluble protein levels

The insoluble protein levels of α-syn were significantly elevated in PD compared to controls in ctx-fg (PD vs. Control 3.4 ± 0.3 vs. 2.3 ± 0.2, F (1.22) = 5.69, *P* = 0.026, Fig. 4B, Table 5). This effect was independent of the *MAPT* haplotype and not present in the ctx-cbl.

**Table 5:** Effects of *MAPT* haplotype and disease status on levels of tau isoforms and α-syn in the insoluble protein fraction in human samples of fusiform gyrus and cerebellum

Protein fraction	Brain region	Two-way ANOVA	Main effect of haplotype	Main effect of disease	Haplotype × disease	Direction of significant effect
		*P* value	*P* value	*P* value	*P* value	
α-syn	ctx-fg	0.134	0.781	**0.026**	0.995	PD > Control
	ctx-cbl	0.953	0.628	0.767	0.875	–
Total tau	ctx-fg	0.150	0.243	0.099	0.587	–
	ctx-cbl	0.576	0.580	0.590	0.229	–
0N3R tau	ctx-fg	0.108	0.252	**0.036**	0.881	PD > Control
	ctx-cbl	0.226	0.301	0.080	0.669	–
0N4R tau	ctx-fg	0.215	0.453	0.098	0.554	–
	ctx-cbl	0.371	0.380	0.145	0.469	–
1N3R tau	ctx-fg	0.362	0.491	0.130	0.889	–
	ctx-cbl	0.385	0.226	0.298	0.375	–
1N4R tau	ctx-fg	0.031	0.062	**0.043**	0.289	PD > Control
	ctx-fg	0.616	0.779	0.199	0.643	–
2N3R tau	ctx-cbl	0.596	0.790	0.303	0.535	–
	ctx-fg	0.966	0.626	0.939	0.992	–
2N4R tau	ctx-cbl	0.564	0.395	0.605	0.287	–
	ctx-fg	0.299	0.079	0.803	0.651	–

Bold values indicate a significant result with *P* < 0.05

Main effect of haplotype was analyzed by comparison of H1/H1 (Controls + PD) versus H2/H2 (Controls + PD). Main effect of disease was analyzed by comparison of PD (H1/H1 + H2/H2) versus Controls (H1/H1 + H2/H2). The interaction haplotype × disease examined the relationship between the independent variables (haplotype + disease) on the dependent variable of protein levels. *P* values were calculated using two-way ANOVA followed by Tukey’s multiple comparisons test

ctx-fg, cortex of fusiform gyrus; ctx-cbl, cerebellum.

**Fig. 5:**
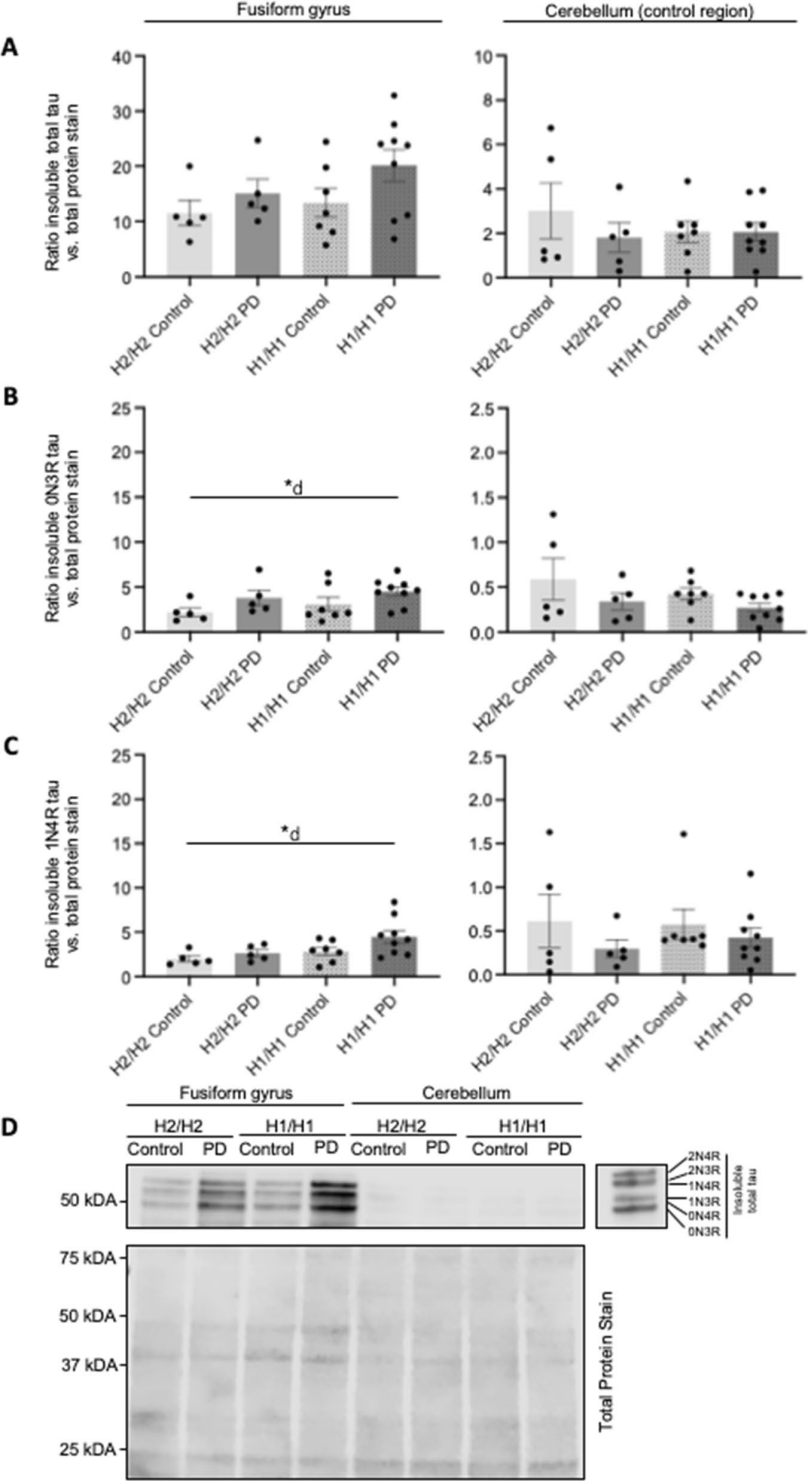
Influence of *MAPT* haplotype and disease status on insoluble tau protein levels. Bar graphs showing the effect of *MAPT* haplotypes (H1/H1, H2/H2) and disease status (PD, control) on insoluble protein levels of total tau (**A**), 0N3R tau (**B**) and 1N4R tau (**C**) in human brain samples of the cortex of fusiform gyrus and the cortex of cerebellum. For analysis of the *MAPT* haplotype effects, PD cases and controls were combined, and vice versa for analysis of the disease status. Data are protein quantity (mean ± SEM) relative to Total Protein Stain and normalized to an inter-run calibration consisting of pooled protein samples. Two-way ANOVA (haplotype × disease status), followed by Tukey’s post-hoc test: *P < 0.05, *d, significant effect of disease. **D** Representative Western blot for soluble total tau with Total Protein Stain as reference. Each group is represented with 1 donor in each blot. The total independent sample size is: H2/H2 Control *n* = 5, H2/H2 PD *n* = 5, H1/H1 Control *n* = 7, H1/H1 PD *n* = 9). The six tau isoforms were identified by comparison to a recombinant tau ladder. Full length Western blots are shown in Additional file 2: Figure S5

The PD brains also had significantly increased insoluble levels of 0N3R and 1N4R tau isoforms compared to controls in ctx-fg (0N3R tau: 4.2 ± 0.4 vs. 2.6 ± 0.5, F (1.22) = 4.99, *P* = 0.036); 1N4R tau: 3.6 ± 0.9 vs. 2.3 ± 0.3, F (1.22) = 4.60, *P* = 0.043), but not in the ctx-cbl (Fig. 5, Table 5). Other tau isoforms (0N4R, 1N3R, 2N3R, 2N4R) did not differ significantly between PD and controls in both brain regions (Table 5). There was a trend towards increased total insoluble tau levels in PD in the ctx-fg, without reaching statistical significance. The proportion of N-terminal and repeat-domain variants of insoluble tau isoforms relative to total insoluble tau were not influenced by *MAPT* haplotype nor disease status (Additional file 1: Table S9). There was no effect of the *MAPT* haplotype or haplotype × disease interaction on insoluble levels of total tau, tau isoforms, or α-syn (Table 5). Also the ratio of the insoluble to soluble protein fractions of tau and α-syn was not influenced by the disease status, the *MAPT* haplotype, or their interaction (Additional file 1: Table S10).

### Protein ratios of fusiform gyrus to cerebellum in human postmortem brains

Despite comparable mRNA expression levels, there was a differential protein ratio with higher insoluble α-syn protein levels in ctx-fg relative to ctx-cbl of PD brains compared to controls (PD vs. Control 2.7 ± 0.3 vs. 1.6 ± 0.2, F (1.22) = 5.72, *P* = 0.025; Fig. 4, Additional file 1: Table S11). The same trend for increased insoluble protein ratios was also observed for insoluble total tau but did not reach statistical significance for the effect of disease (*P* = 0.067*).* However, the ratios of insoluble tau isoform 0N3R (PD vs. Control 3.9 ± 0.4 vs. 2.2 ± 0.5, F (1.22) = 6.58, *P* = 0.018) was significantly higher in PD, independent of the *MAPT* haplotype. For insoluble 1N4R tau protein, a significant increase in H1/H1 PD brains was also found with significant effect of both *MAPT* haplotype (H1/H1 vs. H2/H2 3.2 ± 0.9 vs. 1.9 ± 0.5, F (1,22) = 4.54, *P* = 0.045) and disease (PD vs. Control 3.2 ± 0.8 vs. 1.8 ± 0.4, F (1.22) = 5.12, *P* = 0.034).

## Discussion

Several prior studies have tried to understand the mechanisms underlying the association of the *MAPT* H1/H1 haplotype as a major risk factor for several neurodegenerative diseases, including sporadic PD. So far, studies evaluating the *MAPT* expression in the human brain focused on either healthy controls with regard to *MAPT* haplotypes [34–37] or investigated PD and control cases without consideration of haplotypes [14]. To our knowledge, the present study is the first investigation of *MAPT* regional involvement on gene and protein expression in sporadic PD under the paradigm of different homozygous *MAPT* haplotypes in human brains. Since sporadic PD is classified as synucleinopathy according to its main pathology, we investigated the mRNA and protein levels of the synucleins in the same context. We used ctx-fg as pathological region and ctx-cbl as reference from control brains and PD. Due to the strict selection process, the study group size was rather small, but the included brains were well qualified by their tissue integrity, the severity of PD pathology, and absence of significant concomitant AD pathology.

Our findings support the concept that α-syn pathology in PD does not show up at the transcriptional level or in the soluble protein compartment but is discernible primarily in the insoluble protein compartment. Similar to the study of Quinn et al. [59] we could demonstrate differences on the protein level despite unchanged α-syn mRNA levels. Levels of insoluble α-syn were increased in PD compared to controls independent of the *MAPT* haplotype. This result was observed only in the ctx-fg, but not in ctx-cbl which supports the value of the former as a relevant region of interest for PD. Our findings confirm results of prior postmortem brain studies in which higher levels of insoluble α-syn protein fractions were found in PD compared to controls, while soluble levels of α-syn did not differ, as observed in the amygdala and anterior cingulate gyrus [60], putamen and frontal cortex [61] and in striatum and inferior frontal gyrus [18]. It is worth noting that some prior studies reported a decrease of soluble α-syn levels in PD in cerebellum and occipital cortex [62, 63], yet, no prior data exist on the α-syn protein level in the same protein fractions and brain regions as studied here, making a direct comparison difficult.

Similar to α-syn, tau pathology in PD was also not detectable at the transcriptional level or in the soluble proteome. However, in the insoluble protein compartment, 0N3R and 1N4R tau isoforms were significantly increased in the fusiform gyrus of PD cases. These isoform-specific findings need to be independently reproduced and understood pathophysiologically. Despite various functions, interactions and the potentially crucial role in tau pathology, little is known about the effect of each single exon within the N-terminal domain [64–66]. While for the number of repeats in the C-terminal domain, it is known that compared to 3R tau, 4R tau regulates microtubule dynamics more efficiently, binds with greater affinity to microtubule [67] and is more prone to aggregation [68].

For insoluble total tau, a tendency towards higher levels in PD and H1/H1 was apparent, which did not reach statistical significance due to the small sample size. In contrast to the study of Lei et al., which reported a 44% decrease of soluble levels of tau (phosphate-buffered-saline-soluble) in the substantia nigra in PD compared to controls [69], we did not find significant differences for soluble tau protein. This difference in result may be due to the fact that the SN in PD is strongly degenerated in contrast to the cortex of gyrus fusiformis. Very few studies exist on tau quantity in postmortem human PD brain, however, differences in methodology might explain variable results.

In our study design which allowed a simultanous mRNA and protein extraction from the very same tissue sample, haplotype-specific differences observed at *MAPT* mRNA level were not reflected in total tau protein expression. Only 1N4R tau isoform of insoluble tau showed a trend towards increased expression in the H1/H1 haplotype, but this did not reach the level of statistical significance. Modulating factors involved in alternative splicing of *MAPT* might contribute to the observed discrepancy between *MAPT* mRNA and corresponding tau protein level. The recent work of Bowles et al. 2022 highlighted the role of splicing regulating factors focusing on N-terminal tau splicing. They described differentially altered expression of certain splicing regulating factors and RNA binding proteins by single nuclei gene expression in disease-relevant brain regions of AD and PSP brains compared to controls [3]. Although there are currently no data in PD, such modulating factors might also contribute to the observed discrepancy between *MAPT* RNA and tau protein levels. This might explain the brain region-specific differences in the effects of *MAPT* haplotypes at the protein level and subsequently PD pathogenesis.

As expected, the *MAPT* haplotype, located on chromosome 17, did not influence mRNA expression of *SNCA*, encoded on chromosome 4. The predisposition to PD by the *MAPT* haplotype did not seem to manifest itself at the transcriptional level of the synuclein genes. The lack of influence of disease on expression levels of synuclein genes *SNCA*, *SNCB* and *SNCG* is in good agreement with other postmortem studies. *SNCA* mRNA levels were reported to be increased only in substantia nigra in PD, [40, 70, 71], but not differ in cerebellum or temporal cortex [40, 59] in comparison to controls. To our knowledge, the expression of *SNCB* and *SNCG* in postmortem tissue had so far not been examined in the context of PD, as to our knowledge.

In line with the literature, we found a statistically significant increase of mRNA expression of *total MAPT* in donors with the H1/H1 haplotype [72]. Despite the trend towards higher *MAPT* expression in the cerebellum of H1/H1 *MAPT* haplotype, statistically significant elevated *total MAPT* mRNA levels were only detected in the ctx-fg only, a brain region affected by α-syn pathology. Our results are in agreement with previous studies describing the same association between homozygous *MAPT* haplotypes in frontal cortex and cerebellum of healthy donors [34]. This result was also described in prefrontal cortex of PD cases [73] not stratified for the *MAPT* haplotype. However, there are also studies which could not detect haplotype-specific differences in *total MAPT* expression in healthy human brains. These contradictory findings could result from analysis of groups with heterozygous *MAPT* haplotype status [35] or from different brain regions being examined, since *MAPT* was found to be differently expressed across brain regions [37].

Some studies suggest that the *MAPT* haplotype affects more differential splicing and thus the expression of certain *MAPT* transcripts rather than the overall gene expression [35, 37]. Evidence for this has been provided by postmortem studies of healthy individuals with elevated levels of 4R *MAPT* [36], but lower levels of 2N *MAPT* transcripts for H1/H1 compared to H2/H2 [37]. However, we did not observe a haplotype- or disease-specific influence on the expression of *MAPT* transcripts [35, 74, 75]. To our knowledge, the only study on both mRNA and protein levels in human brain is the large-scale work of Trabzuni et al. which found no clear relationship between *MAPT* mRNA and tau isoforms in healthy donors [37]. The work of Strauß et al. reported the same observation in induced pluripotent stem cells with homozygous *MAPT* haplotype [58]. Given that only few studies exist regarding tau protein stability and its half-life in postmortem brain, this remains difficult to interpret at first glance. Another possible explanation could be the lower stability of RNA compared to protein in postmortem tissue [76].

In contrast to the increased *total MAPT* expression in H1/H1 haplotype, we found *MAPT-AS1* mRNA to be markedly decreased in H1/H1 in the ctx-cbl regardless of disease state. This effect was not observed in ctx-fg. The expression of *MAPT-AS1* has also been studied in postmortem tissue with similar sample size, but only in a comparison of PD versus control. In these studies, a PD-specific decrease in *MAPT-AS1* was found across brain regions with different levels of PD pathology, including SN and cerebellum [40, 41]. Overexpression of the promotor-associated long non-coding RNA *MAPT-AS1* was shown to inhibit *MAPT* promotor activity and *MAPT* expression in human striatal progenitor cells [41]*.* These findings suggest that haplotype-specific regulation of *MAPT* may not be restricted to a specific brain region and could be independent of disease status. Further research is required to gain a deeper understanding of the potential regulatory role of *MAPT-AS1* in the context of PD.

The impact of *MAPT* haplotype on the other genes within the *MAPT* inversion locus remains enigmatic**.** For the other investigated genes neither an effect of the *MAPT* haplotype nor disease was detectable in our study. In contrast to previous studies, in which expression of *PLEKHM1* was found increased in the cerebellum of healthy H1/H1 compared to H2/H2 carriers [34] and increased expression of *STH* was observed in cerebellum of PD patients compared to controls [14]. However, the remaining genes of interest might be addressed in future experiments since differences in mRNA expression play a crucial role in pathologic changes mediated by the *MAPT* haplotypes.

Taken together, our results show that the *MAPT* haplotype influences overall *MAPT* expression and the PD status leads to increased insoluble tau protein in parallel with insoluble α-syn aggregates. This may constitute a risk factor for PD at the biochemical level according to the principle of mutual catalysis of aggregation of tau and α-syn, demonstrated in vitro [19, 20] and in vivo [77]. In all performed analyses, there was no interaction between disease status and *MAPT* haplotype on *MAPT* and *SNCA* gene expression and protein level. All the above observations were detected only in the vulnerable brain region ctx-fg, but not in the control region ctx-cbl, which supports the preferential vulnerability of certain brain regions in PD.

Besides mRNA expression and protein levels itself, the proportion of *MAPT* transcripts to *total MAPT*, respectively tau isoforms to total tau, might also be of relevance. For mRNA, this has already been studied in a few cases in adult frontal cortex only (AD *n* = 3, Control *n* = 3). There the ratio of 3R to 4R *MAPT* was about one, while 1N, 0N, and 2N *MAPT* accounted for 54%, 37% and 9% of *total MAPT* [68, 78]. Despite the lack of significant differences, we observed an increased ratio of 4R to 3R *MAPT* in ctx-fg across all groups. A similar finding was observed in the study of Tobin et al. 2012 in cerebellum in PD compared to healthy donors [68, 78]. No *MAPT* haplotyping was performed in this research publication. With no further reports on splice variants in postmortem human brain, and specifically in ctx-fg, our data contribute to the understanding of *MAPT* splicing, and the distribution of tau isoforms respectively, in the human postmortem brain. Studies with a larger sample size could further investigate and verify the trends observed here.

In addition, we present further data on the regional distribution of tau and α-syn protein across the human brain. In comparison, tau protein levels of both protein fractions were visibly overall higher in ctx-fg compared to ctx-cbl, which has also been demonstrated by other studies [37]. In line with the main analysis, this regional difference was significantly altered in PD cases for insoluble tau isoforms 0N3R and 1N4R tau. Above that, the H1/H1 haplotype was associated with increased insoluble 1N4R tau in ctx-fg. This finding might be linked to the association of *MAPT* haplotype H1/H1 with increased risk of sporadic PD which primarly affects regions besides cerebellum. Still, it is difficult to explain these differences to their full extent, since other insoluble and soluble tau isoforms also showed differences between brain regions but without reaching level of significance. A disease-specific increase was also found for insoluble α-syn levels highlighting the relevance of ctx-fg as a target of study in PD.

The present study is subject to several limitations related to the use of postmortem tissue. First, the limited availability of high-quality brain tissue prevented us from working with larger sample sizes. In addition, it turned out to be challenging to find a sufficient number of donors with H2/H2 *MAPT* haplotype which is less frequent in Europe (5–37.5%) [22, 79]. Secondly, ante- (agonal phase) and postmortem factors (PMI) are suspected to influence postmortem brain tissue quality. For this reason, we selected donors with the shortest PMI and tissue samples with the highest RIN. It is important to mention that the detrimental influence of PMI on mRNA was estimated to be low [80] and the value of RIN as tissue quality marker has been very controversial [81–86]. Third, the ctx-cbl was chosen as reference region in accordance with former post mortem brain studies [34, 87, 88]. Even though LB pathology is typically not present in ctx-cbl, PD related changes have been reported previously indicating a possible involvement of the cerebellum in PD [89]. In turn, the disease-specific findings regarding α-syn in the target brain region confirmed the selection of brain tissue as a validated source for the analysis of *MAPT* haplotype-specific expression differences and the questionable association to PD. With the stringent tissue quality process, we made the best efforts to identify a well-balanced study population in terms of demographic, clinical and neuropathologic parameters. It is important to note that methodological differences (e.g. sample size, brain regions, types of pathologies, or method of quantification) across postmortem brain studies limit the possibility of comparisons among them and with the presented study.

## Conclusion

In summary, we explored the concept of *MAPT* haplotype- and disease-specific gene and protein expression in PD. These findings support the hypothesis that homozygosity for the H1/H1 *MAPT* haplotype predisposes to PD through increased mRNA expression of the *MAPT* gene. This in turn predisposes to increased aggregation of the tau protein, which catalyses the aggregation of α-syn. Future research should address the distinct functions of N- and C-terminal tau domains, the role of transcriptome regulating anti-sense transcripts and the remaining genes located at the 17q21.3 inversion locus in terms of expression, function and potential role in sporadic PD and tauopathies.

## Supplementary Information

**Additional file 1. Table S1**: Title: Demographic, clinical and neuropathologic characteristics of individual postmortem brain donors. Description: The LBD Braak stage and AD Braak and Braak stage each consist of six stages. The CERAD score describes neuritic Amyloid-ß plaques in levels of 0, A, B, C. Lewy inclusion pathology assessed semi-quantitatively the burden of Lewy neurites and Lewy Bodies in the target brain region cortex of fusiform gyrus (+ few, +  + moderate, +  +  + many inclusions). * Since brain stem of this specimen was not available for neuropathological assessment, exclusion of LBD of stage 3 or lower was not possible. Abbreviations: AD, Alzheimer’s disease; bst, brain stem predominant; CERAD, The Consortium to Establish a Registry for Alzheimer’s Disease; C, Control; F, female; CRF, cardiac-respiratory failure; ctx-fg, cortex of fusiform gyrus; CUP, cancer of unknown primary; GI, gastrointestinal; IMP, Imperial College London Brain Bank, LBD, Lewy body disease; limb, limbical; LMU, Neurobiobank of the Ludwig-Maximilians-University of Munich; M, male; n.a. data not available; neo, neocortical; PD, Parkinson’s disease; PMI, postmortem interval; SCLC, small squamous cell lung cancer; CKD, chronic kidney disease; COPD, chronic obstructive pulmonary disease; n.a., not applicable; n.i. no information. **Table S2**: Title: Cause of death of postmortem brain donors. Description: Abbreviations: Fwd, forward; Rev, reverse; bp, base pairs., chronic obstructive pulmonary disease; CKD, chronic kidney disease; n.a., data not available. **Table S3**: Title: Primers for *MAPT* genotyping. Description: Abbreviations: Fwd, forward; Rev, reverse; bp, base pairs. **Table S4**: Title: RNA integrity values measured in postmortem brain samples. Description: For groupwise comparison of the four subgroups according to *MAPT* haplotype and disease *P*-values were calculated using one-way ANOVA. **Table S5**: Title: Primers for qPCR. Description: Abbreviations: Fwd, forward; Rev, reverse; bp, base pairs; *reference gene; † The used primers for the *NSF* gene cover *NSF, NSF* pseudogene and *LRRC37A2*. A detailed description of primer specificity and possible impact on primer usage can be found in the Materials and Methods section under qPCR. **Table S6**: Title: Antibodies used for immunoblotting. Description: Abbreviations: Chemi, chemiluminescence; Rb, rabbit; RT, room temperature. **Table S7**: Title: Proportion of *MAPT* splice variant mRNA transcripts relative to total *MAPT* in conditions defined by *MAPT* haplotype and disease status. Description: For groupwise comparison results were combined according to *MAPT* haplotype and disease status. P-values were calculated using two-way ANOVA followed by Tukey’s multiple comparisons test. Main effect of haplotype was analyzed by comparison of H1/H1 (Controls + PD) versus H2/H2 (Controls + PD). Main effect of disease was analyzed by comparison of PD (H1/H1 + H2/H2) versus Controls (H1/H1 + H2/H2). The interaction haplotype × disease examined the relationship between the independent variables (haplotype + disease) on the dependent variable of proportions of *MAPT* transcripts. Data are mean ± SEM. Statistic analysis were not significant for all parameters (*P* > 0.05). Abbreviations: ctx-fg, cortex of fusiform gyrus; ctx-cbl, cortex of cerebellum. **Table S8**: Title: Expression of candidate mRNAs in human samples of fusiformis gyrus compared to cerebellum. Description: For the comparison of gene expression between the brain regions, the difference in mRNA levels between gyrus fusiformis and cerebellum were calculated. For groupwise comparison of these differences results were combined according to *MAPT* haplotype and disease status. *P*-values were calculated using two-way ANOVA followed by Tukey’s multiple comparisons test and were not significant for all parameters *(P* > 0.05). Abbreviations: ctx-fg, cortex of fusiform gyrus; ctx-cbl, cortex of cerebellum. **Table S9**: Title: N-and R-terminal variants of tau protein isoforms relative to total tau in conditions defined by *MAPT* haplotype and disease status. Description: For groupwise comparison results were combined according to *MAPT* haplotype and disease status. Statistic analysis for groupwise differences with two-way ANOVA were not significant (*P* > 0.05) for all parameters. Abbreviations: ctx-fg, cortex of fusiform gyrus; ctx-cbl, cortex of cerebellum. **Table S10**: Title: Investigation of differences between insoluble and soluble protein levels in human samples of fusiform gyrus and cerebellum. Description: For the comparison of protein levels between the protein fractions, the differences in protein levels between the insoluble and soluble protein fraction were calculated. *P*-values were calculated using two-way ANOVA followed by Tukey’s multiple comparisons test. Statistic analysis were not significant for all parameters (*P* > 0.05). Abbreviations: ctx-fg, cortex of fusiform gyrus; ctx-cbl, cortex of cerebellum. **Table S11**: Title: Regional distribution of different protein levels in human samples of fusiform gyrus related to cerebellum. Description: For the comparison of protein levels between the brain regions, the differences in protein levels between gyrus fusiformis and cerebellum were calculated. For groupwise comparison of these differences results were combined according to *MAPT* haplotype and disease status. *P*-values were calculated using two-way ANOVA followed by Tukey’s multiple comparisons test. Abbreviations: ctx-fg, cortex of fusiform gyrus; ctx-cbl, cortex of cerebellum.

**Additional file 2. Figure S1**: Title: α-synuclein pathology in human postmortem brain tissue. Description: Immunohistochemistry for phosphorylated α-synuclein visualizes exemplary Lewy bodies (LB) (arrows) in cortical cell layers of fusiform gyrus of Parkinson (PD) cases but not of control cases of both *MAPT* haplotypes (H1/H1 and H2/H2). No LB pathology is present in cerebellar cortex of PD cases or control cases of both haplotypes. Purkinje cell layer (p), molecular layer (m) and granular layer (g) Representative images derive from postmortem brain donors with *MAPT* H1/H1 (C2, PD9) and H2/H2 (C9, PD2). Scale bar: 50 µm. **Figure S2**: Title: Full length Western blot from Fig. 3B. Description: (**A**) Lower and (**B**) higher exposed image of the Western blot probed with soluble protein fraction after staining with an antibody against total tau (A0024, DAKO). (**C**) Image of the Western blot after stripping and staining with an antibody against GAPDH (CB1001, Millipore). Representative images derive from postmortem brain donors with *MAPT* H1/H1 (C1, PD5) and H2/H2 (C9, PD14). **Figure S3**: Title: Full length Western blot from Fig. 4C. Description: (**A**) Lower and (**B**) higher exposed image of the Western blot probed with soluble protein fraction after staining with an antibody against α-Syn (2642S, Cell Signaling Technology). (**C**) Image of the Western blot after stripping and staining with an antibody against GAPDH (CB1001, Millipore). Representative images derive from postmortem brain donors with *MAPT* H1/H1 (C1, PD5) and H2/H2 (C9, PD14) on the left side and MAPT H1/H1 (C6, PD4) and H2/H2 (C12, PD10) on the right side. **Figure S4**: Title: Full length Western blot from Fig. 4D. Description: (**A**) Lower and (**B**) higher exposed image of the Western Blot probed with insoluble protein fraction after staining with an antibody against α-Syn (2642S, Cell Signaling Technology). (**C**) Image of the Western blot after stripping and staining with Total Protein Stain (RevertTM 700, LI-COR). Representative images derive from postmortem brain donors with MAPT H1/H1 (C2, PD9) and H2/H2 (C9, PD2) on the left side and *MAPT* H1/H1 (C6, PD4) and H2/H2 (C12, PD10) on the right side. **Figure S5**: Title: Full length Western blot from Fig. 5D. Description: (**A**) Lower and (**B**) higher exposed image of the Western blot probed with insoluble protein fraction after staining with an antibody against total tau (A0024, DAKO). (**C**) Image of the Western blot after stripping and staining with Total Protein Stain (Revert™ 700, LI-COR). Representative images derive from postmortem brain donors with *MAPT* H1/H1 (C1, PD5) and H2/H2 (C9, PD14).

## Data Availability

The data that support the findings of this study are not openly available due to reasons of sensitivity of human data and are available from the corresponding author upon reasonable request.

## Abbreviations

*MAPT*: Microtubule-associated protein tau
PD: Parkinson’s disease
ctx-fg: Cortex of fusiform gyrus
ctx-cbl: Cortex of the cerebellar hemisphere
AD: Alzheimer’s disease
LB: Lewy bodies
α-syn: α-Synuclein
GWAS: Genome-wide association studies
CBD: Corticobasal degeneration
PSP: Progressive supranuclear palsy
LBD: Lewy Body Disease
CERAD score: The Consortium to Establish a Registry for Alzheimer's Disease
PMI: Postmortem interval
RIN: RNA integrity

## References

[1] Raydorsey E, Elbaz A, Nichols E (2018). Global, regional, and national burden of Parkinson’s disease, 1990–2016: a systematic analysis for the Global Burden of Disease Study 2016. Lancet Neurol.

[2] Braak H (2004). Stages in the development of Parkinson's disease-related pathology. Cell Tissue Res.

[3] Houlden H, Singleton AB (2012). The genetics and neuropathology of Parkinson's disease. Acta Neuropathol.

[4] Edwards TL (2010). Genome-wide association study confirms SNPs in SNCA and the MAPT region as common risk factors for Parkinson disease. Ann Hum Genet.

[5] Simon-Sanchez J (2009). Genome-wide association study reveals genetic risk underlying Parkinson's disease. Nat Genet.

[6] Nalls MA (2014). Large-scale meta-analysis of genome-wide association data identifies six new risk loci for Parkinson's disease. Nat Genet.

[7] Lill CM (2016). Genetics of Parkinson's disease. Mol Cell Probes.

[8] Chang D (2017). A meta-analysis of genome-wide association studies identifies 17 new Parkinson's disease risk loci. Nat Genet.

[9] Grenn FP, et al (2020) The Parkinson’s disease GWAS locus browser. bioRxiv, p 2020.04.01.020404

[10] Pascale E (2016). Genetic architecture of MAPT gene region in Parkinson disease subtypes. Front Cell Neurosci.

[11] Lee VM, Goedert M, Trojanowski JQ (2001). Neurodegenerative tauopathies. Annu Rev Neurosci.

[12] Guo T, Noble W, Hanger DP (2017). Roles of tau protein in health and disease. Acta Neuropathol.

[13] Barbier P (2019). Role of tau as a microtubule-associated protein: structural and functional aspects. Front Aging Neurosci.

[14] Tobin JE (2008). Haplotypes and gene expression implicate the MAPT region for Parkinson disease: the GenePD study. Neurology.

[15] Höglinger GU (2017). Differentiation of atypical Parkinson syndromes. J Neural Transm (Vienna).

[16] Colom-Cadena M (2013). Confluence of alpha-synuclein, tau, and beta-amyloid pathologies in dementia with Lewy bodies. J Neuropathol Exp Neurol.

[17] Ishizawa T (2003). Colocalization of tau and alpha-synuclein epitopes in Lewy bodies. J Neuropathol Exp Neurol.

[18] Wills J (2010). Elevated tauopathy and alpha-synuclein pathology in postmortem Parkinson's disease brains with and without dementia. Exp Neurol.

[19] Giasson BI (2003). Initiation and synergistic fibrillization of tau and alpha-synuclein. Science.

[20] Kotzbauer PT (2004). Fibrillization of alpha-synuclein and tau in familial Parkinson's disease caused by the A53T alpha-synuclein mutation. Exp Neurol.

[21] Baker M (1999). Association of an extended haplotype in the tau gene with progressive supranuclear palsy. Hum Mol Genet.

[22] Stefansson H (2005). A common inversion under selection in Europeans. Nat Genet.

[23] Hoglinger GU (2011). Identification of common variants influencing risk of the tauopathy progressive supranuclear palsy. Nat Genet.

[24] Pastor P (2002). Further extension of the H1 haplotype associated with progressive supranuclear palsy. Mov Disord.

[25] Houlden H (2001). Corticobasal degeneration and progressive supranuclear palsy share a common tau haplotype. Neurology.

[26] Kouri N (2015). Genome-wide association study of corticobasal degeneration identifies risk variants shared with progressive supranuclear palsy. Nat Commun.

[27] Myers AJ (2005). The H1c haplotype at the MAPT locus is associated with Alzheimer's disease. Hum Mol Genet.

[28] Pennisi E (2008). Genetics. 17q21.31: not your average genomic address. Science.

[29] Zabetian CP (2007). Association analysis of MAPT H1 haplotype and subhaplotypes in Parkinson's disease. Ann Neurol.

[30] Wider C (2010). Association of the MAPT locus with Parkinson's disease. Eur J Neurol.

[31] Vandrovcova J (2009). Association of MAPT haplotype-tagging SNPs with sporadic Parkinson's disease. Neurobiol Aging.

[32] Nalls MA (2019). Identification of novel risk loci, causal insights, and heritable risk for Parkinson's disease: a meta-analysis of genome-wide association studies. Lancet Neurol.

[33] Zhang CC (2017). Meta-analysis of the association between variants in MAPT and neurodegenerative diseases. Oncotarget.

[34] de Jong S (2012). Common inversion polymorphism at 17q21.31 affects expression of multiple genes in tissue-specific manner. BMC Genom.

[35] Caffrey TM (2006). Haplotype-specific expression of exon 10 at the human MAPT locus. Hum Mol Genet.

[36] Caffrey TM, Joachim C, Wade-Martins R (2008). Haplotype-specific expression of the N-terminal exons 2 and 3 at the human MAPT locus. Neurobiol Aging.

[37] Trabzuni D (2012). MAPT expression and splicing is differentially regulated by brain region: relation to genotype and implication for tauopathies. Hum Mol Genet.

[38] Ni Y (2017). Investigation of long non-coding RNA expression profiles in the Substantia Nigra of Parkinson's disease. Cell Mol Neurobiol.

[39] Xin C, Liu J (2021). Long non-coding RNAs in Parkinson's disease. Neurochem Res.

[40] Elkouris M (2019). Long non-coding RNAs associated with neurodegeneration-linked genes are reduced in Parkinson’s disease patients. Front Cell Neurosci.

[41] Coupland KG (2016). Role of the long non-coding RNA MAPT-AS1 in regulation of microtubule associated protein tau (MAPT) expression in Parkinson's disease. PLoS ONE.

[42] Cheng WW, Zhu Q, Zhang HY (2020). Identifying risk genes and interpreting pathogenesis for Parkinson's disease by a multiomics analysis. Genes (Basel).

[43] Liu X (2011). Genome-wide association study identifies candidate genes for Parkinson's disease in an Ashkenazi Jewish population. BMC Med Genet.

[44] Burré J (2010). Alpha-synuclein promotes SNARE-complex assembly in vivo and in vitro. Science.

[45] Gubas A (2021). The endolysosomal adaptor PLEKHM1 is a direct target for both mTOR and MAPK pathways. FEBS Lett.

[46] Braak H (2003). Staging of brain pathology related to sporadic Parkinson's disease. Neurobiol Aging.

[47] Braak H, Braak E (1991). Neuropathological stageing of Alzheimer-related changes. Acta Neuropathol.

[48] Mirra SS (1991). The Consortium to Establish a Registry for Alzheimer's Disease (CERAD) Part II. Standardization of the neuropathologic assessment of Alzheimer's disease. Neurology.

[49] McKeith IG (2017). Diagnosis and management of dementia with Lewy bodies: Fourth consensus report of the DLB Consortium. Neurology.

[50] Greene JG (2012). Current status and future directions of gene expression profiling in Parkinson's disease. Neurobiol Dis.

[51] Simunovic F (2010). Evidence for gender-specific transcriptional profiles of nigral dopamine neurons in Parkinson disease. PLoS ONE.

[52] Hellemans J (2007). qBase relative quantification framework and software for management and automated analysis of real-time quantitative PCR data. Genome Biol.

[53] Spicakova T (2010). Expression and silencing of the microtubule-associated protein Tau in breast cancer cells. Mol Cancer Ther.

[54] Fagerberg L (2014). Analysis of the human tissue-specific expression by genome-wide integration of transcriptomics and antibody-based proteomics. Mol Cell Proteom.

[55] Sun M (2021). Systematic functional interrogation of human pseudogenes using CRISPRi. Genome Biol.

[56] Cabana-Domínguez J (2016). A highly polymorphic copy number variant in the NSF gene is associated with cocaine dependence. Sci Rep.

[57] Sudmant PH (2010). Diversity of human copy number variation and multicopy genes. Science.

[58] Strauß T (2021). iPS cell-based model for MAPT haplotype as a risk factor for human tauopathies identifies no major differences in TAU expression. Front Cell Dev Biol.

[59] Quinn JG (2012). alpha-Synuclein mRNA and soluble alpha-synuclein protein levels in post-mortem brain from patients with Parkinson's disease, dementia with Lewy bodies, and Alzheimer's disease. Brain Res.

[60] Yamasaki TR (2019). Parkinson's disease and multiple system atrophy have distinct α-synuclein seed characteristics. J Biol Chem.

[61] Zhou J (2011). Changes in the solubility and phosphorylation of α-synuclein over the course of Parkinson's disease. Acta Neuropathol.

[62] Quinn JG (2012). α-Synuclein mRNA and soluble α-synuclein protein levels in post-mortem brain from patients with Parkinson's disease, dementia with Lewy bodies, and Alzheimer's disease. Brain Res.

[63] Westerlund M (2008). Cerebellar alpha-synuclein levels are decreased in Parkinson's disease and do not correlate with SNCA polymorphisms associated with disease in a Swedish material. Faseb J.

[64] Horowitz PM (2006). N-terminal fragments of tau inhibit full-length tau polymerization in vitro. Biochemistry.

[65] McKibben K, Rhoades E (2019) Regulation of tau’s proline rich region by its N-terminal domain*.* bioRxiv, p 633420 10.1074/jbc.RA119.010172 PMC6916478 31699899

[66] Jeganathan S (2006). Global hairpin folding of tau in solution. Biochemistry.

[67] Rosenberg KJ (2008). Complementary dimerization of microtubule-associated tau protein: implications for microtubule bundling and tau-mediated pathogenesis. Proc Natl Acad Sci USA.

[68] Goedert M (1989). Multiple isoforms of human microtubule-associated protein tau: sequences and localization in neurofibrillary tangles of Alzheimer's disease. Neuron.

[69] Lei P (2012). Tau deficiency induces parkinsonism with dementia by impairing APP-mediated iron export. Nat Med.

[70] Simunovic F (2009). Gene expression profiling of substantia nigra dopamine neurons: further insights into Parkinson's disease pathology. Brain.

[71] Gründemann J (2008). Elevated alpha-synuclein mRNA levels in individual UV-laser-microdissected dopaminergic substantia nigra neurons in idiopathic Parkinson's disease. Nucleic Acids Res.

[72] Rademakers R (2005). High-density SNP haplotyping suggests altered regulation of tau gene expression in progressive supranuclear palsy. Hum Mol Genet.

[73] Valenca GT (2016). The role of MAPT haplotype H2 and isoform 1N/4R in Parkinsonism of older adults. PLoS ONE.

[74] Myers AJ (2007). The MAPT H1c risk haplotype is associated with increased expression of tau and especially of 4 repeat containing transcripts. Neurobiol Dis.

[75] Majounie E (2013). Variation in tau isoform expression in different brain regions and disease states. Neurobiol Aging.

[76] Ferrer I (2008). Brain banks: benefits, limitations and cautions concerning the use of post-mortem brain tissue for molecular studies. Cell Tissue Bank.

[77] Masliah E (2001). beta-amyloid peptides enhance alpha-synuclein accumulation and neuronal deficits in a transgenic mouse model linking Alzheimer's disease and Parkinson's disease. Proc Natl Acad Sci U S A.

[78] Brandt R, Hundelt M, Shahani N (2005). Tau alteration and neuronal degeneration in tauopathies: mechanisms and models. Biochim Biophys Acta.

[79] Donnelly MP (2010). The distribution and most recent common ancestor of the 17q21 inversion in humans. Am J Hum Genet.

[80] Robinson AC (2016). Extended post-mortem delay times should not be viewed as a deterrent to the scientific investigation of human brain tissue: a study from the Brains for Dementia Research Network Neuropathology Study Group, UK. Acta Neuropathol.

[81] Sonntag KC (2016). Limited predictability of postmortem human brain tissue quality by RNA integrity numbers. J Neurochem.

[82] Imbeaud S (2005). Towards standardization of RNA quality assessment using user-independent classifiers of microcapillary electrophoresis traces. Nucleic Acids Res.

[83] Becker C (2010). mRNA and microRNA quality control for RT-qPCR analysis. Methods.

[84] Fleige S (2006). Comparison of relative mRNA quantification models and the impact of RNA integrity in quantitative real-time RT-PCR. Biotechnol Lett.

[85] Koppelkamm A (2011). RNA integrity in post-mortem samples: influencing parameters and implications on RT-qPCR assays. Int J Legal Med.

[86] Fleige S, Pfaffl MW (2006). RNA integrity and the effect on the real-time qRT-PCR performance. Mol Aspects Med.

[87] Chappell S (2018). Observations of extensive gene expression differences in the cerebellum and potential relevance to Alzheimer's disease. BMC Res Notes.

[88] Negi SK, Guda C (2017). Global gene expression profiling of healthy human brain and its application in studying neurological disorders. Sci Rep.

[89] Wu T, Hallett M (2013). The cerebellum in Parkinson's disease. Brain.

