# Supplementary material for: Different MAPT haplotypes influence expression of total MAPT in postmortem brain tissue
Source: Acta Neuropathol Commun. 2023 Mar 11;11:40. doi: 10.1186/s40478-023-01534-9 (PMC10008602; doi:10.1186/s40478-023-01534-9)
Supplement: Supplementary file 1 — Additional file 1. Table S1: Title: Demographic, clinical and neuropathologic characteristics of individual postmortem brain donors. Description: The LBD Braak stage and AD Braak and Braak stage each consist of six stages. The CERAD score describes neuritic Amyloid-ß plaques in levels of 0, A, B, C. Lewy inclusion pathology assessed semi-quantitatively the burden of Lewy neurites and Lewy Bodies in the target brain region cortex of fusiform gyrus (+ few, + + moderate, + + + many inclusions). * Since brain stem of this specimen was not available for neuropathological assessment, exclusion of LBD of stage 3 or lower was not possible. Abbreviations: AD, Alzheimer’s disease; bst, brain stem predominant; CERAD, The Consortium to Establish a Registry for Alzheimer’s Disease; C, Control; F, female; CRF, cardiac-respiratory failure; ctx-fg, cortex of fusiform gyrus; CUP, cancer of unknown primary; GI, gastrointestinal; IMP, Imperial College London Brain Bank, LBD, Lewy body disease; limb, limbical; LMU, Neurobiobank of the Ludwig-Maximilians-University of Munich; M, male; n.a. data not available; neo, neocortical; PD, Parkinson’s disease; PMI, postmortem interval; SCLC, small squamous cell lung cancer; CKD, chronic kidney disease; COPD, chronic obstructive pulmonary disease; n.a., not applicable; n.i. no information. Table S2: Title: Cause of death of postmortem brain donors. Description: Abbreviations: Fwd, forward; Rev, reverse; bp, base pairs., chronic obstructive pulmonary disease; CKD, chronic kidney disease; n.a., data not available. Table S3: Title: Primers for MAPT genotyping. Description: Abbreviations: Fwd, forward; Rev, reverse; bp, base pairs. Table S4: Title: RNA integrity values measured in postmortem brain samples. Description: For groupwise comparison of the four subgroups according to MAPT haplotype and disease P-values were calculated using one-way ANOVA. Table S5: Title: Primers for qPCR. Description: Abbreviations: Fwd, forward; Rev, reverse; bp, base pa [file 40478_2023_1534_MOESM1_ESM.docx]

**Table S1. Demographic, clinical and neuropathologic characteristics of individual postmortem brain donors.**

| **Brain donor**  **(Sex)** | **Age at diagnosis**  **(y)** | **Age at death**  **(y)** | **Disease duration**  **(y)** | **PMI**  **(h)** | **LBD Braak stage** | **Lewy inclusion pathology in ctx-fg** | **LBD**  **McKeith stage** | **AD Braak and Braak stage** | **CERAD score** |
| --- | --- | --- | --- | --- | --- | --- | --- | --- | --- |
| PD1 (F) | 68 | 79 | 11 | 17 | 6 | ++ | neo | II | 0 |
| PD2 (F) | 58 | 72 | 14 | 12 | 5 | + | limb/neo | I | 0 |
| PD3 (F) | n.a. | 76 | n.a. | 12 | 6 | +++ | limb | II | 0 |
| PD4 (M) | n.a. | 74 | n.a. | n.a. | >5 | + | n.a. | I | 0 |
| PD5 (M) | 69 | 72 | 3 | 28 | 6 | ++ | neo | I | 0 |
| PD6 (M) | 73 | 74 | 2 | 14 | 6 | ++ | neo | III | 0 |
| PD7 (F) | 55 | 79 | 24 | 9 | 6 | + | neo | II | B |
| PD8 (M) | 66 | 72 | 6 | 19 | 4 | - | pre | II | 0 |
| PD 9 (M) | 61 | 70 | 7 | 17 | 6 | + | neo | I | 0 |
| PD10 (F) | 67 | 82 | 15 | n.a. | 6 | + | neo | I | 0 |
| PD11 (F) | 78 | 85 | 7 | 29 | 6 | +++ | neo | I | 0 |
| PD12 (M) | 68 | 78 | 10 | 11 | 6 | - | neo | I | A |
| PD13 (M) | 59 | 66 | 7 | 14 | 6 | + | pre | I | 0 |
| PD14 (F) | n.a. | 77 | n.a. | 9 | >4 | + | n.a. | II | A |
| C1 (F) | - | 77 | - | 8 | * | - | * | II | 0 |
| C2 (F) | - | 73 | - | 14 | 0 | - | - | II | 0 |
| C3 (F) | - | 66 | - | n.a. | 0 | - | - | II | 0 |
| C4 (M) | - | 74 | - | 28 | 0 | - | - | I | 0 |
| C5 (M) | - | 65 | - | 12 | 0 | - | - | I | 0 |
| C6 (F) | - | 84 | - | 11 | 0 | - | - | II | 0 |
| C7 (M) | - | 79 | - | 21 | 0 | - | - | I | 0 |
| C8 (F) | - | 60 | - | 15 | 0 | - | - | I | 0 |
| C9 (M) | - | 70 | - | 40 | 0 | - | - | I | 0 |
| C10 (F) | - | 89 | - | 22 | 0 | - | - | I | 0 |
| C11 (F) | - | 95 | - | 28 | 0 | - | - | III | 0 |
| C12 (F) | - | 91 | - | 22 | 0 | - | - | I | 0 |
| The postmortem brain donors PD1-PD9 and C1-C7 carried the MAPT haplotype H1/H1, the donors PD10-PD14 and C8-C12 carried H2/H2. The LBD Braak stage and AD Braak and Braak stage each consist of six stages. The CERAD score describes neuritic Amyloid-ß plaques in levels of 0, A, B, C. Lewy inclusion pathology assessed semi-quantitatively the burden of Lewy neurites and Lewy Bodies in the target brain region cortex of fusiform gyrus. * Since brain stem of this specimen was not available for neuropathological assessment, exclusion of LBD of stage 3 or lower was not possible. Abbreviations: AD, Alzheimer´s disease; CERAD, The Consortium to Establish a Registry for Alzheimer´s Disease;C, Control; F, female; ctx-fg, cortex of fusiform gyrus; LBD, Lewy body disease; limb, limbical; M, male; n.a. data not available; neo, neocortical; PD, Parkinson´s disease; PMI, postmortem interval; pre, brain stem predominant; + few, ++ moderate, +++ many inclusions. | | | | | | | | | |

**Table S2. Cause of death of postmortem brain donors.**

| **Participant**  **number** | Controls | | PD | |
| --- | --- | --- | --- | --- |
|  | **H1/H1**  (*n* = 7) | **H2/H2**  (*n* = 5) | **H1/H1**  (*n* = 9) | **H2/H2**  (*n* = 5) |
| 1 | CRF | Exacerbated COPD | CRF | GI bleeding |
| 2 | Sepsis | n.a. | Ovarial carcinoma | Lung cancer |
| 3 | Pneumonia | n.a. | Pulmonary embolism | Pneumonia |
| 4 | Cardiac arrest | Pneumonia, CKD | CRF | Metastatic CUP |
| 5 | SCLC | Pneumonia | Cancer bleeding | Aneurysm rupture |
| 6 | Pancreatic cancer | - | Pneumonia | - |
| 7 | Pneumonia | - | Pneumonia | - |
| 8 | - | - | Pneumonia | - |
| 9 | - | - | n.a. | - |
| Abbreviations: CRF, cardiac-respiratory failure; GI, gastrointestinal; CUP, cancer of unknown primary; SCLC, small squamous cell lung cancer; COPD, chronic obstructive pulmonary disease; CKD, chronic kidney disease; n.a., data not available. | | | | |

Table S3. Primers for *MAPT* genotyping.

| Primer | Primer pair sequence (5’-3’) forward/reverse | Product size (bp) |
| --- | --- | --- |
| *MAPT*_rs8070723 | Fwd TAATAGCAAAGCCCCAGTTGTC | 299 |
|  | Rev CAGTGATGAACCCAAGCTCCT |  |
| *MAPT*_rs17650901 | Fwd CGGCCAACTGTTAGAGAGGG | 361 |
|  | Rev CTGGATGCAAACTGTTCCCG |  |
| *MAPT*_rs1052553 | Fwd CTTCCACCTGCCTAACCCAG | 400 |
|  | Rev CCAGCCACTCTCACCTTCCC |  |
| *MAPT*_rs9468 | Fwd AACCCACAAGCTGACCTTCC | 371 |
|  | Rev GCCAAAGCCGAGTGACAAAAG |  |
| Abbreviations: Fwd, forward; Rev, reverse; bp, base pairs. | | |

**Table S4. RNA integrity values measured in postmortem brain samples.**

| **Brain region** | **Participant**  **number** | **Controls** | | **PD** | | **Groupwise comparison**  ***P*–value** |
| --- | --- | --- | --- | --- | --- | --- |
|  |  | **H1/H1**  **(*n* = 7)** | **H2/H2**  **(*n* = 5)** | **H1/H1**  **(*n* = 9)** | **H2/H2**  **(*n* = 5)** |  |
| Cortex of fusiform gyrus | 1 | 8.8 | 6.1 | 5.1 | 5.8 |  |
|  | 2 | 6.4 | 6.5 | 6.4 | 3.1 |  |
|  | 3 | 5.7 | 2.8 | 5.5 | 4.8 |  |
|  | 4 | 5.5 | 5.6 | 5.0 | 6.7 |  |
|  | 5 | 5.9 | 6.5 | 6.1 | 6.4 |  |
|  | 6 | 4.4 | - | 4.5 | - |  |
|  | 7 | 6.2 | - | 8.5 | - |  |
|  | 8 | - | - | 6.6 | - |  |
|  | 9 | - | - | 6.5 | - |  |
|  | Mean (SEM) | 6.1 (0.5) | 5.5 (0.7) | 6.0 (0.4) | 5.4 (0.7) | 0.704 |
| Cortex of cerebellum | 1 | 8.3 | 3.2 | 3.2 | 5.6 |  |
|  | 2 | 7.4 | 7.0 | 7.0 | 2.8 |  |
|  | 3 | 5.5 | 4.6 | 4.6 | 5.1 |  |
|  | 4 | 5.0 | 4.6 | 4.6 | 2.8 |  |
|  | 5 | 4.9 | 5.5 | 5.5 | 6.1 |  |
|  | 6 | 4.4 | - | - | - |  |
|  | 7 | 6.8 | - | - | - |  |
|  | 8 | - | - | - | - |  |
|  | 9 | - | - | - | - |  |
|  | Mean (SEM) | 6.1 (0.6) | 5.0 (0.6) | 5.0 (0.6) | 4.5 (0.7) | 0.107 |
| For groupwise comparison of the four subgroups according to *MAPT* haplotype and disease *P*-values were calculated using one-way ANOVA. | | | | | | |

Table S5. Primers for qPCR.

| Gene | Description | Primer pair sequence (5’-3’) Forward/Reverse | Product size (bp) |
| --- | --- | --- | --- |
| *GAPDH** | Glyeraldehyde-3-phosphate  dehydrogenase | Fwd TCGGAGTCAACGGATTTGGT | 195 |
|  |  | Rev CCTGGAAGATGGTGATGGGA |  |
| *ACTIN** | Actin beta | Fwd TCACCAACTGGGACGACATG | 208 |
|  |  | Rev GAGGCGTACAGGGATAGCAC |  |
| *TBP** | TATA-box binding protein | Fwd AAAGAACGCTGTACTCAGTGTG | 155 |
|  |  | Rev CCCCGGTTGAGGGCTTTTA |  |
| *Total MAPT* | Microtubule  associated protein tau | Fwd GAGTCCAGTCGAAGATTGGGT | 223 |
|  |  | Rev GGCGAGTCTACCATGTCGATG |  |
| *MAPT 3R* | Microtubule-associated  protein tau, 3-repeats | Fwd AGGCGGGAAGGTGCAAATAG | 104 |
|  |  | Rev CCTGGCCACCTCCTGGTTTATG |  |
| *MAPT 4R* | Microtubule*-a*ssociated  protein tau, 4-repeats | Fwd GCCCATGCCAGACCTGAAGA | 165 |
|  |  | Rev CCTCCCGGGACGTGTTTGAT |  |
| *MAPT 0N* | Microtubule-associated  protein tau, 0 N-terminal inserts | Fwd GCTGGCCTGAAAGCTGAAG | 120 |
|  |  | Rev ATCGCTTCCAGTCCCGTCT |  |
| *MAPT 1N* | Microtubule-associated  protein tau, 1 N-terminal inserts | Fwd CAACAGCGGAAGCTGAAGAA | 68 |
|  |  | Rev GTGACCAGCAGCTTCGTCTT |  |
| *MAPT 2N* | Microtubule-associated  protein tau, 2 N-terminal inserts | Fwd ACTCCAACAGCGGAAGATGT | 159 |
|  |  | Rev GTGACCAGCAGCTTCGTCTT |  |
| *MAPT-AS1* | Microtubule*-a*ssociated  protein tau antisense RNA 1 | Fwd AGATGCACCTGCAGCCC | 121 |
|  |  | Rev CCCGTCCTTGTTCTGACTCC |  |
| *NSF****^†^*** | N-ethylmaleimide sensitive  factor, vesicle fusing ATPase | Fwd GCTGGGCTTTCTATTGGGCAA | 100 |
|  |  | Rev GCAGGAAATCAATCTCGATGGT |  |
| *PLEKHM1* | Pleckstrin homology and  run domaining containing M1 | Fwd GGAGTCCCCCTGTAAGAAGC | 132 |
|  |  | Rev GCTGGTAGTTTCCTCACCCA |  |
| *STH* | Saitohin | Fwd ACAGAACCCTCAGCTTAGCAT | 161 |
|  |  | Rev GCCTTCAATGGAAAGTTGTCTTC |  |
| *SNCA* | Alpha-synuclein | Fwd AAGAGGGTGTTCTCTATGTAGGC | 106 |
|  |  | Rev GCTCCTCCAACATTTGTCACTT |  |
| *SNCB* | Beta-synuclein | Fwd GGGCTCAATCAGTGGTTCTT | 91 |
|  |  | Rev CACAGGACTGGTGAAGAGGG |  |
| *SNCG* | Gamma-synuclein | Fwd CACAGGACTGGTGAAGAGGG | 148 |
|  |  | Rev GGAGAACATCGCGGTCAC |  |
| Abbreviations: Fwd, forward; Rev, reverse; bp, base pairs; *reference gene; **^†^ The used primers for the NSF gene cover NSF, NSF pseudogene and LRRC37A2. A detailed description of primer specificity and possible impact on primer usage can be found in the Materials and Methods section under qPCR.** | | | |

Table S6. Antibodies used for immunoblotting.

| **Antibody** | Incubation conditions | Characteristic (host) | Settings for imaging  (Channel- min) | Manufacturer  (Reference number) |
| --- | --- | --- | --- | --- |
| Primary antibodies |  |  |  |  |
| α-syn | 1:500  (24h, 4 °C) | Polyclonal (rb) | 600 – 1 min  700 – 1 min  Chemi – 20 min | Cell Signaling Technology  (2642S) |
| Total tau | 1:3000  (24 h, 4 °C) | Polyclonal (rb) | 600 **–** 1 min,  700 – 1 min  Chemi - 15 min | DAKO  (A0024) |
| GAPDH | 1:3000  (24 h, 4 °C) | Monoclonal (rb) | 600 – 1 min  700 – 1 min  Chemi – 8 min | Millipore  (CB1001) |
| Secondary antibodies |  |  |  |  |
| Peroxidase | 1:5000  (2h, RT) | Anti-rb (goat) | - | Millipore  (PI1000) |
| Abbreviations: Chemi, chemiluminescence; Rb, rabbit; RT, room temperature. | | | | |

Table S7. Proportion of *MAPT* splice variant mRNA transcripts relative to total *MAPT* in conditions defined by *MAPT* haplotype and disease status.

| ***MAPT* transcript** | Controls | | PD | | Two-way ANOVA | | |
| --- | --- | --- | --- | --- | --- | --- | --- |
|  | H1/H1 | H2/H2 | H1/H1 | H2/H2 | **Main effect of haplotype**  *P*-value | **Main effect of disease**  *P*-value | **Haplotype**  **× disease**  *P*-value |
| ctx-fg | | | | | | | |
| 0N / total MAPT (%) | 35 ± 18 | 42 ± 18 | 35 ± 17 | 50 ± 42 | 0.664 | 0.865 | 0.865 |
| 1N / total MAPT (%) | 32 ± 20 | 24 ± 14 | 40 ± 17 | 4 ± 3 | 0.230 | 0.743 | 0.427 |
| 2N / total MAPT (%) | 33 ± 32 | 34 ± 33 | 25 ± 22 | 46 ± 43 | 0.721 | 0.954 | 0.754 |
| ctx-cbl | | | | | | | |
| 0N / total MAPT (%) | 31 ± 7 | 29 ± 8 | 34 ± 2 | 29 ± 5 | 0.592 | 0.794 | 0.729 |
| 1N / total MAPT (%) | 28 ± 4 | 29 ± 8 | 36 ± 2 | 28 ± 7 | 0.536 | 0.538 | 0.447 |
| 2N / total MAPT (%) | 41 ± 10 | 42 ± 11 | 30 ± 3 | 43 ± 4 | 0.426 | 0.553 | 0.453 |
| ctx-fg | | | | | | | |
| 3R / total MAPT (%) | 35 ± 31 | 42 ± 19 | 34 ± 29 | 34 ± 31 | 0.910 | 0.902 | 0.921 |
| 4R / total MAPT (%) | 65 ± 50 | 58 ± 20 | 66 ± 18 | 66 ± 31 | 0.907 | 0.898 | 0.919 |
| ctx-cbl | | | | | | | |
| 3R / total MAPT (%) | 45 ± 18 | 90 ± 34 | 54 ± 14 | 47 ± 22 | 0.823 | 0.870 | 0.829 |
| 4R / total MAPT (%) | 55 ± 47 | 10 ± 9 | 46 ± 12 | 53 ± 28 | 0.610 | 0.496 | 0.473 |
| For groupwise comparison results were combined according to *MAPT* haplotype and disease status. *P*-values were calculated using two-way ANOVA followed by Tukey´s multiple comparisons test. Main effect of haplotype was analyzed by comparison of H1/H1 (Controls + PD) vs. H2/H2 (Controls + PD). Main effect of disease was analyzed by comparison of PD (H1/H1+H2/H2) vs Controls (H1/H1+H2/H2). The interaction haplotype × disease examined the relationship between the independent variables (haplotype + disease) on the dependent variable of proportions of *MAPT* transcripts. Data are mean ± SEM. Statistic analysis were not significant for all parameters (*P* > 0.05). Abbreviations: ctx-fg, cortex of fusiform gyrus; ctx-cbl, cortex of cerebellum. | | | | | | | |

**Table S8. Expression of candidate mRNAs in human samples of fusiformis gyrus compared to cerebellum.**

| **Transcript** | **mRNA levels ctx-fg vs. ctx-cbl**  **Mean ± SEM** | | | | Two-way ANOVA | | |
| --- | --- | --- | --- | --- | --- | --- | --- |
|  | Controls | | PD | |  |  |  |
|  | H1/H1 | H2/H2 | H1/H1 | H2/H2 | **Main effect**  **of haplotype**  *P*-value | **Main effect of disease**  *P*-value | **Haplotype**  **× disease**  *P*-value |
| *Total MAPT* | -0.04 ± 0.16 | 0.15 ± 0.32 | 0.17 ± 0.14 | -0.40 ± 0.18 | 0.342 | 0.415 | 0.067 |
| *0N MAPT* | -0.33 ± 0.35 | 0.48 ± 0.25 | -0.02 ± 0.11 | 0.01 ± 0.09 | 0.296 | 0.748 | 0.112 |
| *1N MAPT* | -0.14 ± 0.22 | 0.25 ± 0.32 | 0.06 ± 0.13 | -0.15 ± 0.20 | 0.667 | 0.643 | 0.172 |
| *2N MAPT* | -0.62 ± 0.52 | 0.42 ± 0.53 | 0.44 ± 0.20 | 0.34 ± 0.18 | 0.245 | 0.220 | 0.162 |
| *3R MAPT* | -0.09 ± 0.44 | 0.21 ± 0.25 | 0.36 ± 0.09 | 0.13 ± 0.11 | 0.895 | 0.512 | 0.349 |
| *4R MAPT* | 0.14 ± 0.41 | 0.43 ± 0.37 | 0.43 ± 0.37 | 0.24 ± 0.28 | 0.823 | 0.593 | 0.264 |
| *MAPT-AS1* | 0.29 ± 0.35 | -0.39 ± 0.30 | 0.25 ± 0.12 | -0.47 ± 0.21 | 0.182 | 0.876 | 0.953 |
| *STH* | 0.13 ± 0.15 | 0.01 ± 0.32 | -0.02 ± 0.11 | -0.15 ± 0.23 | 0.438 | 0.513 | 0.978 |
| *PLEKHM1* | 0.27 ± 0.21 | -0.08 ± 0.27 | -0.06 ± 0.17 | -0.19 ± 0.22 | 0.285 | 0.336 | 0.616 |
| *NSF* | 0.07 ± 0.18 | -0.05 ± 0.21 | 0.06 ± 0.13 | -0.15 ± 0.2 | 0.343 | 0.763 | 0.789 |
| *SNCA* | 0.15 ± 0.16 | 0..06 ± 0.21 | -0.06 ± 0.10 | -0.16 ± 0.23 | 0.589 | 0.208 | 0.998 |
| *SNCB* | 0.24 ± 0.31 | 0.41 ± 0.35 | -0.56 ± 0.38 | 0.27 ± 0.10 | 0.177 | 0.204 | 0.372 |
| *SNCG* | 0.03 ± 0.18 | 0.18 ± 0.36 | -0.14 ± 0.20 | 0.03 ± 0.30 | 0.541 | 0.530 | 0.946 |
| For the comparison of gene expression between the brain regions, the difference in mRNA levels between gyrus fusiformis and cerebellum were calculated. For groupwise comparison of these differences results were combined according to *MAPT* haplotype and disease status. *P*-values were calculated using two-way ANOVA followed by Tukey´s multiple comparisons test and were not significant for all parameters (*P* > 0.05). Abbreviations: ctx-fg, cortex of fusiform gyrus; ctx-cbl, cortex of cerebellum. | | | | | | | |

Table S9. N-and R-terminal variants of tau protein isoforms relative to total tau in conditions defined by *MAPT* haplotype and disease status.

| **Tau isoform** | Controls | | PD | | Two-way ANOVA | | |
| --- | --- | --- | --- | --- | --- | --- | --- |
|  | H1/H1 | H2/H2 | H1/H1 | H2/H2 | **Main effect**  **of haplotype**  *P*-value | **Main effect of disease**  *P*-value | **Haplotype**  **× disease**  *P*-value |
| Soluble tau | | | | | | | |
| ctx-fg |  |  |  |  |  |  |  |
| 0N / total tau (%) | 33 ± 12 | 23 ± 6 | 37 ± 8 | 35 ± 4 | 0.553 | 0.413 | 0.653 |
| 1N / total tau (%) | 34 ± 14 | 22 ± 9 | 37 ± 7 | 35 ± 7 | 0.519 | 0.487 | 0.641 |
| 2N/ total tau (%) | 33 ± 15 | 55 ± 26 | 26 ± 8 | 30 ± 5 | 0.375 | 0.293 | 0.522 |
| ctx-cbl |  |  |  |  |  |  |  |
| 0N/ total tau (%) | 43 ± 8 | 32 ± 8 | 25 ± 3 | 32 ± 8 | 0.977 | 0.291 | 0.127 |
| 1N/ total tau (%) | 42 ± 8 | 33 ± 9 | 36 ± 11 | 34 ± 7 | 0.599 | 0.800 | 0.779 |
| 2N/ total tau (%) | 15 ± 13 | 35 ± 18 | 39 ± 11 | 34 ± 16 | 0.556 | 0.419 | 0.383 |
| ctx-fg |  |  |  |  |  |  |  |
| 3R/ total Tau (%) | 48 ± 19 | 64 ± 19 | 45 ± 11 | 45 ± 4 | 0.669 | 0.556 | 0.686 |
| 4R/ total Tau (%) | 52 ± 22 | 36 ± 12 | 55 ± 12 | 55 ± 10 | 0.626 | 0.502 | 0.645 |
| ctx-cbl |  |  |  |  |  |  |  |
| 3R/ total Tau (%) | 64 ± 13 | 48 ± 13 | 41 ± 9 | 31 ± 22 | 0.368 | 0.158 | 0.847 |
| 4R/ total Tau (%) | 36 ± 12 | 52 ± 11 | 59 ± 16 | 75 ± 12 | 0.286 | 0.137 | 0.957 |
| Insoluble tau | | | | | | | |
| ctx-fg |  |  |  |  |  |  |  |
| 0N / total tau (%) | 40 ± 9 | 38 ±7 | 42 ± 4 | 45 ± 11 | 0.951 | 0.647 | 0.766 |
| 1N / total tau (%) | 42 ± 8 | 38 ± 7 | 42 ± 5 | 40 ± 10 | 0.893 | 0.692 | 0.841 |
| 2N/ total tau (%) | 18 ± 4 | 24 ± 5 | 16 ± 3 | 15 ± 4 | 0.563 | 0.198 | 0.393 |
| ctx-cbl |  |  |  |  |  |  |  |
| 0N/ total tau (%) | 34 ± 5 | 39 ± 16 | 28 ± 5 | 36 ± 11 | 0.423 | 0.589 | 0.860 |
| 1N/ total tau (%) | 39 ± 9 | 45 ± 16 | 38 ± 9 | 39 ± 10 | 0.745 | 0.707 | 0.846 |
| 2N/ total tau (%) | 27 ± 4 | 16 ± 5 | 34 ± 7 | 25 ± 9 | 0.169 | 0.295 | 0.830 |
| ctx-fg | | | | | | | |
| 3R/ total Tau (%) | 52 ± 10 | 51 ± 9 | 50 ± 7 | 58 ± 11 | 0.713 | 0.763 | 0.671 |
| 4R/ total Tau (%) | 48 ± 9 | 49 ± 10 | 50 ± 8 | 42 ± 7 | 0.694 | 0.748 | 0.650 |
| ctx-cbl |  |  |  |  |  |  |  |
| 3R/ total Tau (%) | 48 ± 6 | 55 ± 16 | 48 ± 10 | 59 ± 13 | 0.445 | 0.864 | 0.845 |
| 4R/ total Tau (%) | 52 ± 11 | 45 ± 14 | 52 ± 10 | 41 ± 13 | 0.518 | 0.885 | 0.869 |
| For groupwise comparison results were combined according to *MAPT* haplotype and disease status. Statistic analysis for groupwise differences with two-way ANOVA were not significant (*P* > 0.05) for all parameters. Abbreviations: ctx-fg, cortex of fusiform gyrus; ctx-cbl, cortex of cerebellum. | | | | | | | |

**Table S10. Investigation of differences between insoluble and soluble protein levels in human samples of fusiform gyrus and cerebellum.**

| **Protein**  **(Brain region)** | **Insolube vs. soluble protein levels**  **Mean ± SEM** | | | | Two-way ANOVA | | |
| --- | --- | --- | --- | --- | --- | --- | --- |
|  | Controls | | PD | |  |  |  |
|  | H1/H1 | H2/H2 | H1/H1 | H2/H2 | **Main effect of haplotype**  *P*-value | **Main effect of disease**  *P*-value | **Haplotype**  **× disease**  *P*-value |
| α-syn  (ctx-fg) | -0.87 ± 0.44 | 0.59 ± 0.46 | 1.67 ± 0.46 | 1.44 ± 0.51 | 0.610 | 0.112 | 0.970 |
| α-syn (ctx-cbl) | 0.07 ± 0.18 | 0.10 ± 0.42 | 0.15 ± 0.17 | 0.17 ± 0.35 | 0.932 | 0.787 | 0.990 |
| Total tau (ctx-fg) | 0.72 ± 5.54 | 3.68 ± 2.77 | 7.29 ± 4.21 | 6.13 ± 2.56 | 0.846 | 0.337 | 0.658 |
| Total tau (ctx-cbl) | 0.82 ± 0.63 | 0.22 ± 1.03 | 0.06 ± 0.64 | 0.79 ± 0.70 | 0.935 | 0.900 | 0.309 |
| 0N3R tau (ctx-fg) | 0.84 ± 1.11 | 1.17 ± 0.52 | 1.95 ± 0.80 | 2.08 ± 0.85 | 0.808 | 0.293 | 0.912 |
| 0N3R tau (ctx-cbl) | 0.15 ± 0.04 | 0.16 ± 0.20 | 0.01 ± 0.04 | 0.08 ± 0.07 | 0.698 | 0.244 | 0.722 |
| 0N4R tau (ctx-fg) | 0.39 ± 0.81 | 1.44 ± 0.66 | 1.79 ± 0.79 | 1.61 ± 0.87 | 0.610 | 0.365 | 0.474 |
| 0N4R tau (ctx-cbl) | 0.11 ± 0.06 | 0.13 ± 0.18 | 0.00 ± 0.07 | 0.01 ± 0.11 | 0.905 | 0.270 | 0.937 |
| 1N3R tau (ctx-fg) | 0.87 ± 0.71 | 1.53 ± 0.54 | 1.94 ± 0.68 | 2.04 ± 1.15 | 0.641 | 0.333 | 0.728 |
| 1N3R tau (ctx-cbl) | 0.11 ± 0.05 | 0.31 ± 0.29 | 0.05 ± 0.11 | 0.10 ± 0.14 | 0.418 | 0.372 | 0.634 |
| 1N4R tau (ctx-fg) | 0.36 ± 1.13 | 1.05 ± 0.40 | 1.82 ± 1.02 | 0.89 ± 0.42 | 0.906 | 0.526 | 0.427 |
| 1N4R tau (ctx-cbl) | 0.31 ± 0.14 | 0.12 ± 0.31 | -0.07 ± 0.25 | -0.11 ± 0.19 | 0.647 | 0.219 | 0.749 |
| 2N3R tau (ctx-fg) | -0.90 ± 0.95 | -1.83 ± 2.35 | 0.53 ± 0.76 | 0.59 ± 0.79 | 0.724 | 0.130 | 0.691 |
| 2N3R tau (ctx-cbl) | 0.27 ± 0.10 | -0.16 ± 0.31 | 0.03 ± 0.17 | -0.07 ± 0.32 | 0.237 | 0.724 | 0.459 |
| 2N4R tau (ctx-fg) | -0.84 ± 1.08 | 0.30 ± 0.58 | -0.74 ± 0.74 | -1.08 ± 0.74 | 0.649 | 0.478 | 0.413 |
| 2N4R (ctx-cbl) | 0.48 ± 0.11 | -0.37 ± 0.25 | -0.15 ± 0.14 | 0.07 ± 0.21 | 0.089 | 0.557 | 0.946 |
| For the comparison of protein levels between the protein fractions, the differences in protein levels between the insoluble and soluble protein fraction were calculated. *P*-values were calculated using two-way ANOVA followed by Tukey´s multiple comparisons test. Statistic analysis were not significant for all parameters (*P* > 0.05). Abbreviations: ctx-fg, cortex of fusiform gyrus; ctx-cbl, cortex of cerebellum. | | | | | | | |

T**able S11. Regional distribution of different protein levels in human samples of fusiform gyrus related to cerebellum.**

| **Protein** | **Protein levels ctx-fg vs ctx-cbl**  **Mean ± SEM** | | | | Two-way ANOVA | | |
| --- | --- | --- | --- | --- | --- | --- | --- |
|  | Controls | | PD | |  |  |  |
|  | H1/H1 | H2/H2 | H1/H1 | H2/H2 | **Main effect of haplotype**  *P*-value | **Main effect of disease**  *P*-value | **Haplotype**  **× disease**  *P*-value |
| Soluble α-syn | 0.92 ± 0.29 | 1.02 ± 0.18 | 1.29 ± 0.34 | 1.28 ± 0.30 | 0.894 | 0.350 | 0.861 |
| Insoluble α-syn | 1.72 ± 0.38 | 1.52 ± 0.20 | 2.82 ± 0.48 | 2.54 ± 0.40 | 0.601 | 0.025 | 0.936 |
| Soluble tau | | | | | | | |
| Total tau | 11.7 ± 5.12 | 5.08 ± 4.17 | 10.6 ± 3.08 | 7.93 ± 1.51 | 0.255 | 0.827 | 0.613 |
| 0N3R tau | 1.98 ± 0.79 | 0.59 ± 0.30 | 2.29 ± 0.54 | 1.47 ± 0.22 | 0.082 | 0.334 | 0.646 |
| 0N4R tau | 1.68 ± 0.78 | 0.33 ± 0.29 | 1.88± 0.48 | 1.10 ± 0.16 | 0.076 | 0.410 | 0.620 |
| 1N3R tau | 1.61 ± 0.70 | 0.35 ± 0.29 | 1.78 ± 0.43 | 1.06 ± 0.16 | 0.068 | 0.407 | 0.608 |
| 1N4R tau | 2.21 ± 1.04 | 0.46 ± 0.41 | 2.15 ± 0.69 | 1.35 ± 0.31 | 0.122 | 0.607 | 0.557 |
| 2N3R tau | 1.90 ± 0.87 | 2.73 ± 2.51 | 0.77 ± 0.80 | 0.54 ± 0.86 | 0.812 | 0.194 | 0.673 |
| 2N4R tau | 2.34 ± 0.97 | 0.59 ± 0.70 | 1.79 ± 0.50 | 1.7 ± 0.75 | 0.239 | 0.714 | 0.287 |
| Insoluble tau | | | | | | | |
| Total tau | 11.6 ± 2.72 | 8.54 ± 2.86 | 17.8 ± 2.76 | 13.2 ± 1.95 | 0.190 | 0.067 | 0.791 |
| 0N3R tau | 2.67 ± 0.76 | 1.61 ± 0.57 | 4.23 ± 0.50 | 3.47 ± 0.75 | 0.186 | 0.018 | 0.824 |
| 0N4R tau | 1.96 ± 0.42 | 1.64 ± 0.78 | 3.66 ± 0.66 | 2.70 ± 0.79 | 0.362 | 0.057 | 0.644 |
| 1N3R tau | 2.37 ± 0.59 | 1.57 ± 0.72 | 3.68 ± 0.61 | 3.00 ± 1.01 | 0.322 | 0.075 | 0.935 |
| 1N4R tau | 2.27 ± 0.48 | 1.39 ± 0.54 | 4.04 ± 0.66 | 2.34 ± 0.32 | 0.045 | 0.034 | 0.500 |
| 2N3R tau | 0.73 ± 0.19 | 1.07 ± 0.17 | 1.27 ± 0.33 | 1.20 ± 0.47 | 0.693 | 0.310 | 0.536 |
| 2N4R tau | 1.02 ± 0.34 | 1.26 ± 0.35 | 1.20 ± 0.49 | 0.56 ± 0.39 | 0.661 | 0.572 | 0.341 |
| For the comparison of protein levels between the brain regions, the differences in protein levels between gyrus fusiformis and cerebellum were calculated. For groupwise comparison of these differences results were combined according to *MAPT* haplotype and disease status. *P*-values were calculated using two-way ANOVA followed by Tukey´s multiple comparisons test. Abbreviations: ctx-fg, cortex of fusiform gyrus; ctx-cbl, cortex of cerebellum. | | | | | | | |
