# Supplementary material for: Different MAPT haplotypes influence expression of total MAPT in postmortem brain tissue
Source: Acta Neuropathol Commun. 2023 Mar 11;11:40. doi: 10.1186/s40478-023-01534-9 (PMC10008602; doi:10.1186/s40478-023-01534-9)
Supplement: Supplementary file 2 — Additional file 2. Figure S1: Title: α-synuclein pathology in human postmortem brain tissue. Description: Immunohistochemistry for phosphorylated α-synuclein visualizes exemplary Lewy bodies (LB) (arrows) in cortical cell layers of fusiform gyrus of Parkinson (PD) cases but not of control cases of both MAPT haplotypes (H1/H1 and H2/H2). No LB pathology is present in cerebellar cortex of PD cases or control cases of both haplotypes. Purkinje cell layer (p), molecular layer (m) and granular layer (g) Representative images derive from postmortem brain donors with MAPT H1/H1 (C2, PD9) and H2/H2 (C9, PD2). Scale bar: 50 µm. Figure S2: Title: Full length Western blot from Fig. 3B. Description: (A) Lower and (B) higher exposed image of the Western blot probed with soluble protein fraction after staining with an antibody against total tau (A0024, DAKO). (C) Image of the Western blot after stripping and staining with an antibody against GAPDH (CB1001, Millipore). Representative images derive from postmortem brain donors with MAPT H1/H1 (C1, PD5) and H2/H2 (C9, PD14). Figure S3: Title: Full length Western blot from Fig. 4C. Description: (A) Lower and (B) higher exposed image of the Western blot probed with soluble protein fraction after staining with an antibody against α-Syn (2642S, Cell Signaling Technology). (C) Image of the Western blot after stripping and staining with an antibody against GAPDH (CB1001, Millipore). Representative images derive from postmortem brain donors with MAPT H1/H1 (C1, PD5) and H2/H2 (C9, PD14) on the left side and MAPT H1/H1 (C6, PD4) and H2/H2 (C12, PD10) on the right side. Figure S4: Title: Full length Western blot from Fig. 4D. Description: (A) Lower and (B) higher exposed image of the Western Blot probed with insoluble protein fraction after staining with an antibody against α-Syn (2642S, Cell Signaling Technology). (C) Image of the Western blot after stripping and staining with Total Protein Stain (RevertTM 700, LI-COR). Representative image [file 40478_2023_1534_MOESM2_ESM.pdf]

SUPPL FIG 1

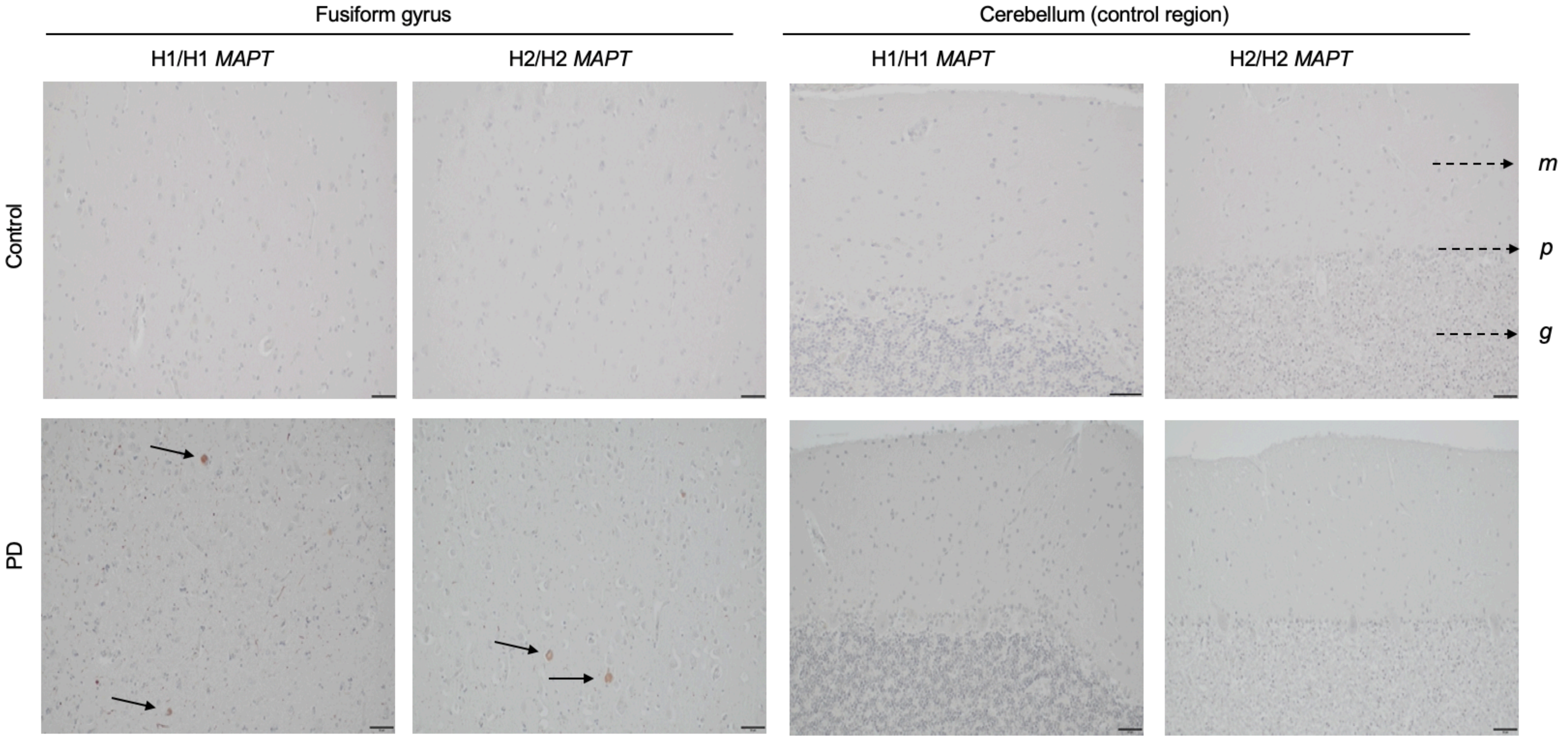
Fusiform gyrus
Cerebellum (control region)
H1/H1 *MAPT*
H2/H2 *MAPT*
H1/H1 *MAPT*
H2/H2 *MAPT*
Control
PD
m
p
g

SUPPL FIG 2

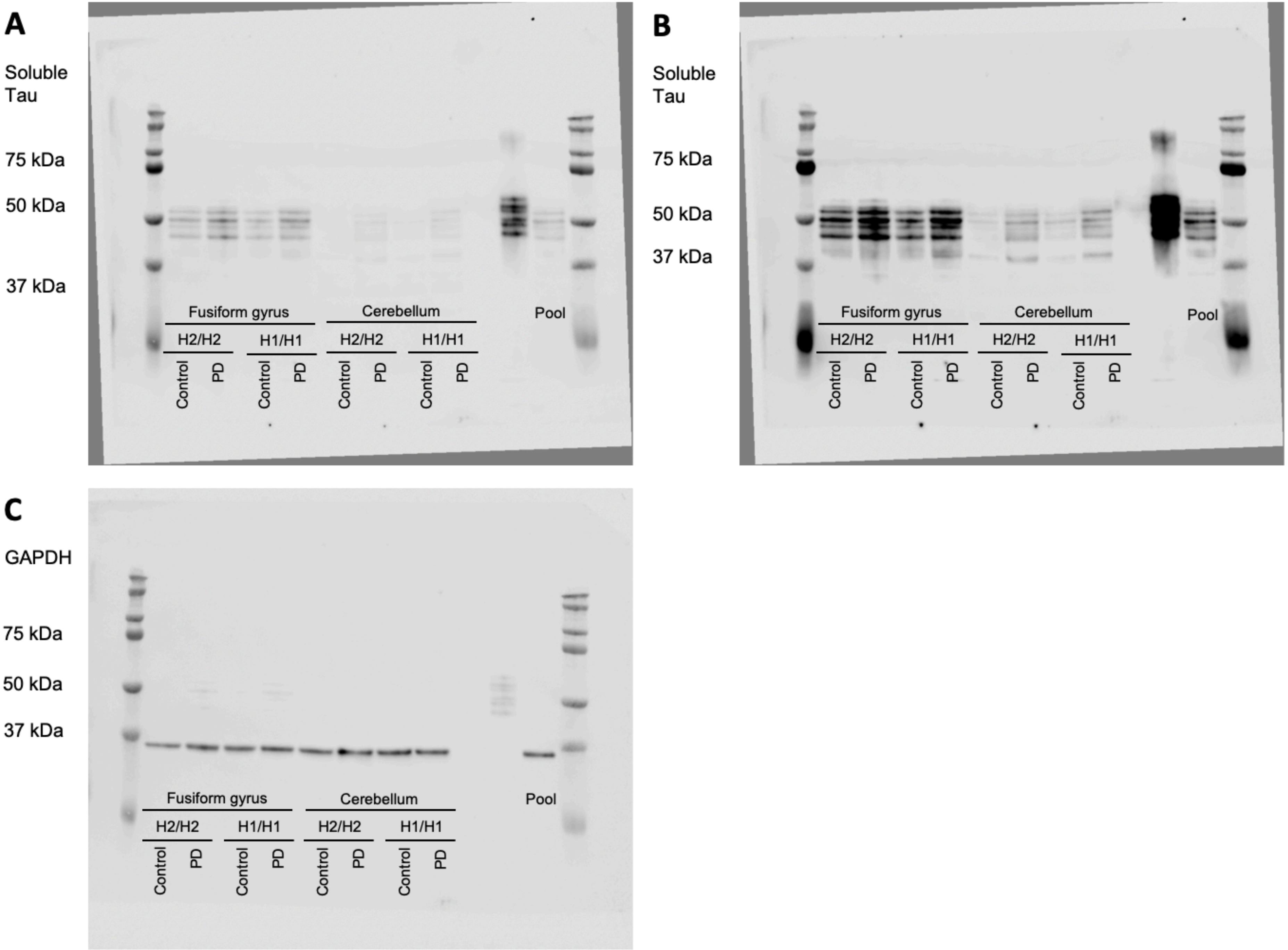
A
Soluble Tau
75 kDa
50 kDa
37 kDa
Fusiform gyrus
Cerebellum
Pool
H2/H2
H1/H1
H2/H2
H1/H1
Control
PD
Control
PD
Control
PD
Control
PD
B
Soluble Tau
75 kDa
50 kDa
37 kDa
Fusiform gyrus
Cerebellum
Pool
H2/H2
H1/H1
H2/H2
H1/H1
Control
PD
Control
PD
Control
PD
Control
PD
C
GAPDH
75 kDa
50 kDa
37 kDa
Fusiform gyrus
Cerebellum
Pool
H2/H2
H1/H1
H2/H2
H1/H1
Control
PD
Control
PD
Control
PD
Control
PD

SUPPL FIG 3

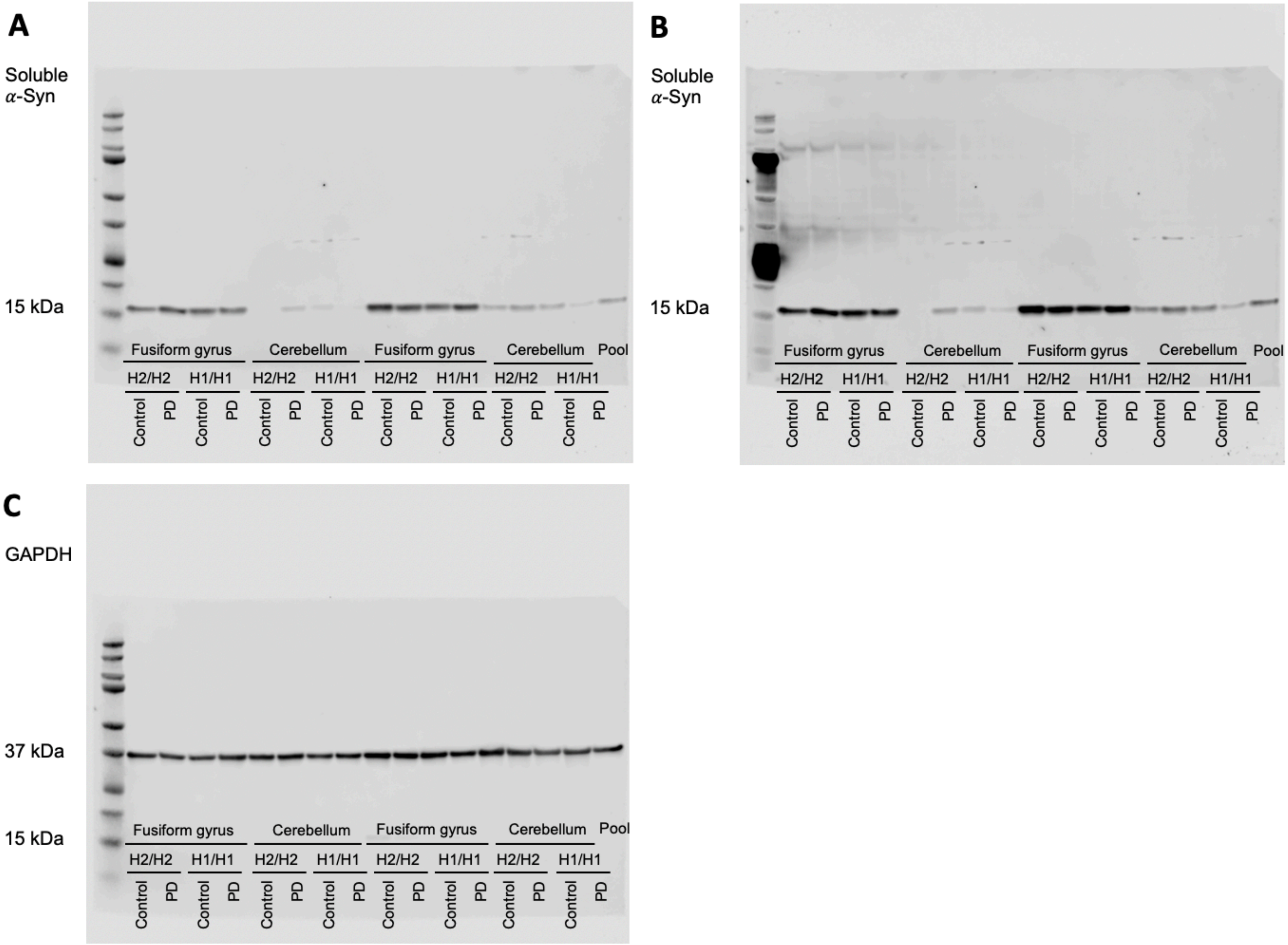
A
Soluble α-Syn
15 kDa
Fusiform gyrus
Cerebellum
Fusiform gyrus
Cerebellum
Pool
H2/H2
H1/H1
H2/H2
H1/H1
H2/H2
H1/H1
H2/H2
H1/H1
Control
PD
B
Soluble α-Syn
15 kDa
Fusiform gyrus
Cerebellum
Fusiform gyrus
Cerebellum
Pool
H2/H2
H1/H1
Control
PD
C
GAPDH
37 kDa
15 kDa
Fusiform gyrus
Cerebellum
Fusiform gyrus
Cerebellum
Pool
H2/H2
H1/H1
Control
PD

SUPPL FIG 4

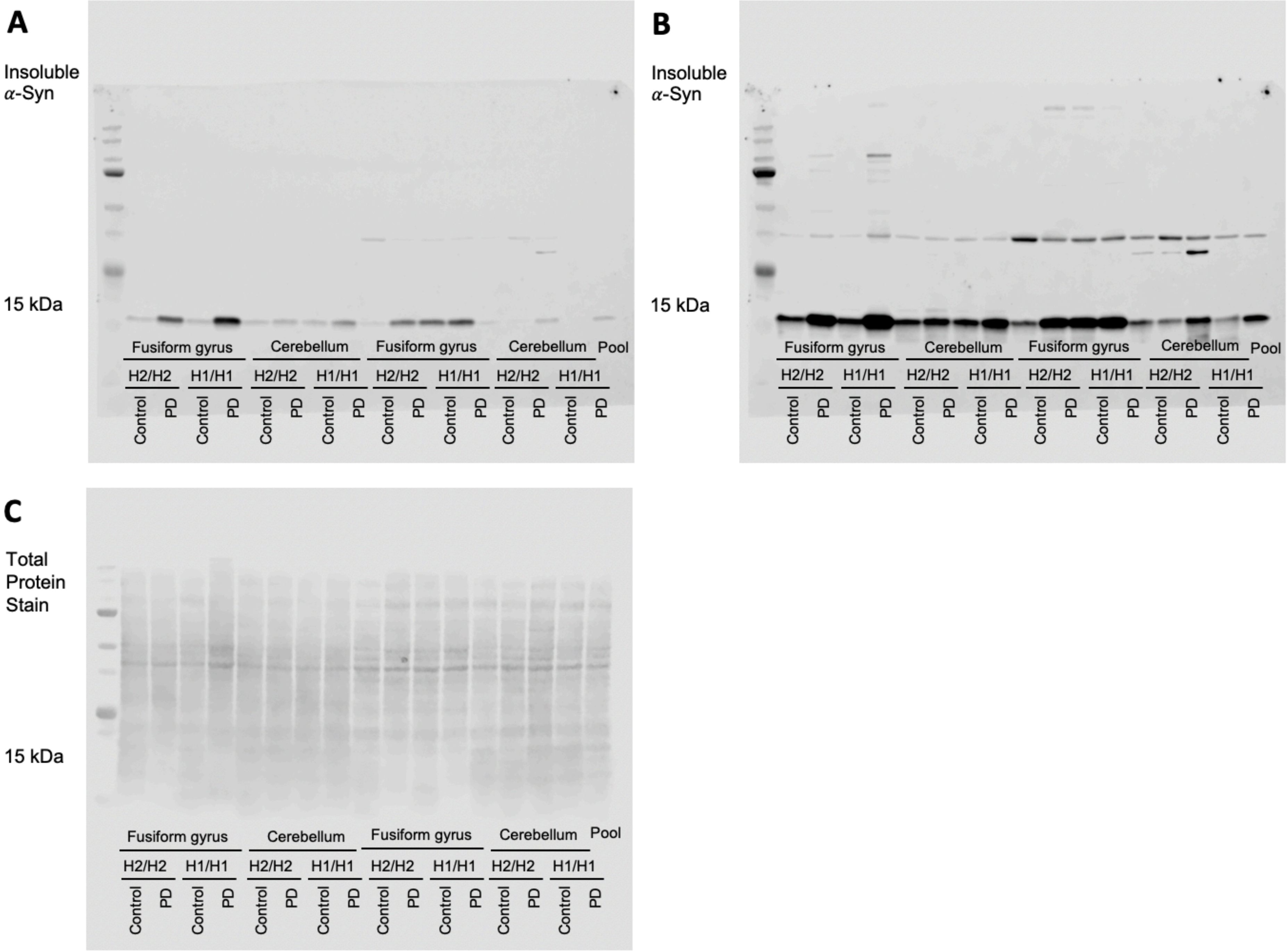
A
Insoluble
α-Syn
15 kDa
Fusiform gyrus
Cerebellum
Fusiform gyrus
Cerebellum
Pool
H2/H2
H1/H1
Control
PD
B
Insoluble
α-Syn
15 kDa
C
Total
Protein
Stain
15 kDa

SUPPL FIG 5

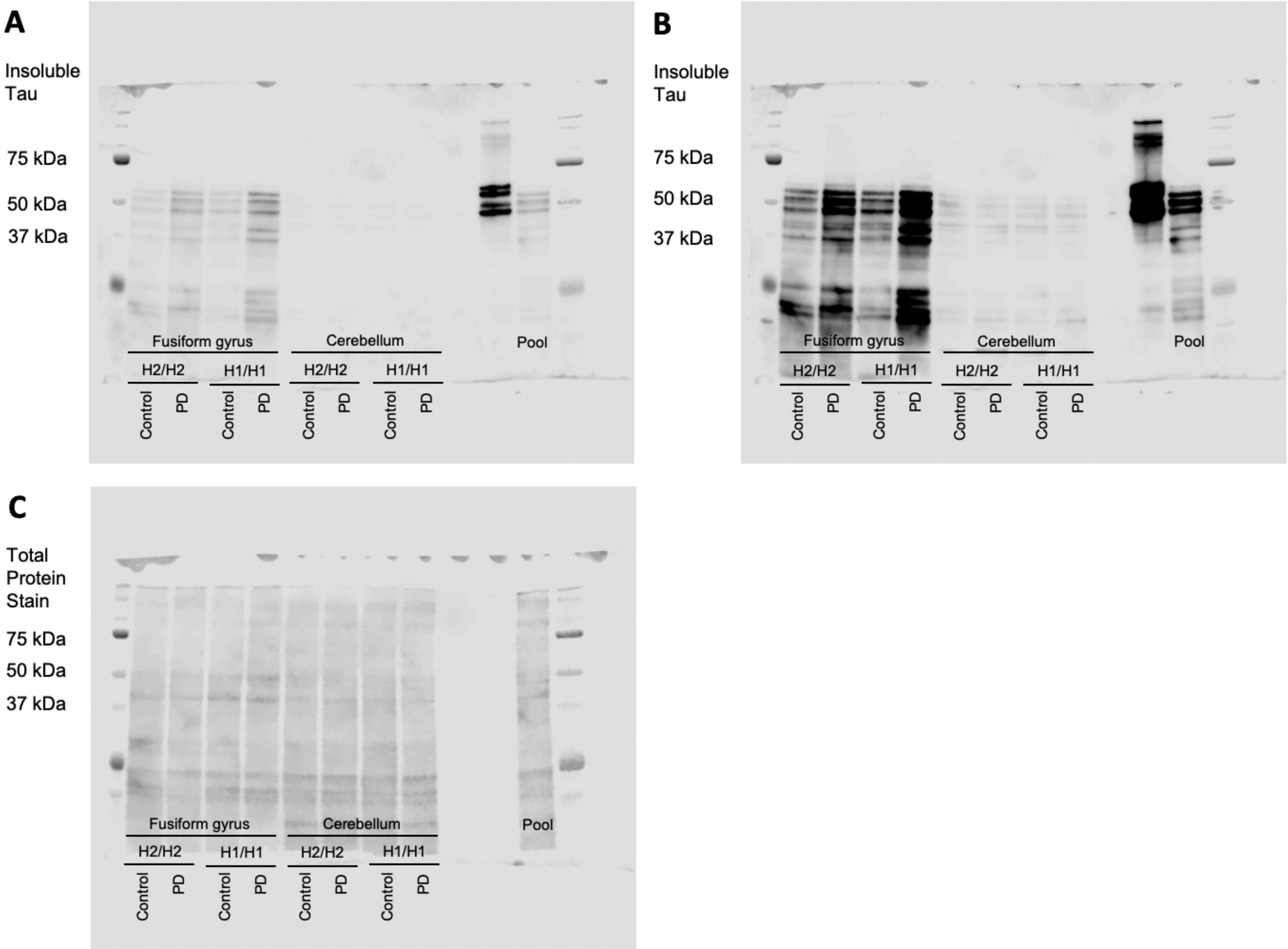
A
Insoluble Tau
75 kDa
50 kDa
37 kDa
Fusiform gyrus
Cerebellum
Pool
H2/H2
H1/H1
H2/H2
H1/H1
Control
PD
Control
PD
Control
PD
Control
PD
B
Insoluble Tau
75 kDa
50 kDa
37 kDa
Fusiform gyrus
Cerebellum
Pool
H2/H2
H1/H1
H2/H2
H1/H1
Control
PD
Control
PD
Control
PD
Control
PD
C
Total Protein Stain
75 kDa
50 kDa
37 kDa
Fusiform gyrus
Cerebellum
Pool
H2/H2
H1/H1
H2/H2
H1/H1
Control
PD
Control
PD
Control
PD
Control
PD
